# Microbiome and metabolic disruption in acute vs. severe and enduring anorexia nervosa

**DOI:** 10.1038/s41522-025-00847-y

**Published:** 2025-11-26

**Authors:** Petra Prochazkova, Janet Jezkova, Radka Roubalova, Katerina Zadakova, Kristyna Coufalova, Gabriela Kubisova, Jakub Kreisinger, Jaroslav Semerad, Alena Nehasilova, Tomas Cajthaml, Helena Tlaskalova-Hogenova, Petra Holanova, Alena Lambertova, Hana Papezova

**Affiliations:** 1 Institute of Microbiology of the Czech Academy of Sciences, Prague 4, Czech Republic; 2 First Faculty of Medicine, Charles University, Prague 2, Czech Republic; 3 Faculty of Science, Department of Zoology, Charles University, Prague 2, Czech Republic; 4 Institute for Environmental Studies, Faculty of Science, Charles University, Prague 2, Czech Republic; 5 Department of Psychiatry, First Faculty of Medicine, Charles University and General University Hospital in Prague, Prague 2, Czech Republic

**Keywords:** Microbiology, Health care

## Abstract

Anorexia nervosa (AN) is associated with profound alterations in gut microbiota and host metabolic profiles. While previous studies have primarily focused on the acute phase of AN, the chronic form, severe and enduring anorexia nervosa (SEAN), remains underexplored in terms of microbiome dynamics. In this study, we characterized gut microbiota composition (via 16S rRNA gene amplicon sequencing), serum and fecal metabolites (via mass spectrometry), and an extensive range of clinical, anthropometric, biochemical, and psychiatric parameters in females with acute AN, SEAN, and in healthy controls. SEAN patients exhibited higher antidepressant usage and greater lifetime stress exposure. Acute AN patients presented with more pronounced eating disorder severity and depressive symptoms. Elevated levels of intestinal fatty acid-binding protein in SEAN patients suggest mucosal damage. Microbiota analysis revealed reduced alpha diversity and distinct community composition in both AN groups, with SEAN showing the greatest interindividual variability. Both AN cohorts exhibited significantly lower serum and fecal γ-aminobutyric acid (GABA) levels, which were negatively correlated with taxa such as *Christensenellaceae*, *Ruminococcaceae*, and *Escherichia-Shigella*, i.e., microorganisms potentially associated with GABA degradation or impaired synthesis. Additionally, reductions in short-chain fatty acids suggest impaired microbial fermentation and dysregulation of the gut-brain axis. Collectively, these findings reveal progressive, functionally relevant changes in microbiota-host interactions in SEAN. These alterations likely reflect the persistent disease state and may contribute to its continuation.

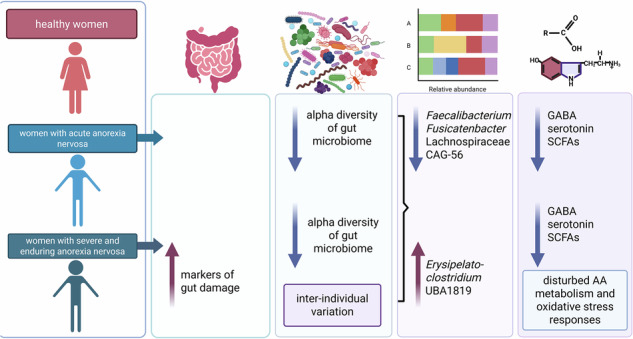

## Introduction

Anorexia nervosa (AN) is a severe psychiatric disorder characterized by extreme dietary restriction, compulsive exercise, and significantly low body weight, often leading to profound malnutrition. Individuals affected typically present with gastrointestinal, cardiovascular, immunological, and metabolic disturbances, along with frequent psychiatric comorbidities. Resistance to treatment further complicates both early intervention and long-term recovery.

The extended duration of this disease allows for the delineation of its phases into acute anorexia and severe enduring anorexia nervosa (SEAN)^1^. Key features of SEAN include a chronic course lasting over seven years, persistent food restriction, underweight status, distorted weight and body shape perception, and completion of at least two medical treatments (e.g., tube feeding or intravenous interventions) alongside a diagnostic evaluation. SEAN is notably resistant to conventional therapies and is associated with poor clinical outcomes.

Growing evidence implicates gut microbiota dysbiosis in a wide range of diseases, including autoimmune and neurodegenerative disorders, as well as cancer. In AN, the gut microbiome has emerged as a potentially significant factor in disease progression and recovery. The gut-brain axis, through which microbiota modulate neural, endocrine, and immune signaling, may contribute to the complex symptomatology of AN.

Despite growing interest in microbiome research related to AN, most studies concentrate on the acute phase, with limited or no data available on SEAN. Given the chronic nature and treatment resistance of SEAN, it is plausible that disruptions in gut microbiota are more pronounced. Exploring these long-term microbial alterations could provide insights into disease chronification and open avenues for novel interventions, including nutritional strategies, emotion-focused therapies, and microbiome-targeted approaches such as fecal microbiota transplantation.

Most studies examining the gut microbiota in patients with AN report significant alterations in both composition and diversity. Although specific bacterial taxa findings vary across studies, a consistent pattern of reduced alpha diversity has been observed. This reduction is primarily attributed to restricted caloric intake and a limited variety of diet^2–7^.

Indigestible food components, particularly dietary fiber, are metabolized by gut microbes to produce short-chain fatty acids (SCFAs), including acetate, propionate, and butyrate. These metabolites serve as energy sources that support intestinal barrier integrity and modulate host metabolism. SCFAs signal through the vagus nerve and, notably, can cross the blood-brain barrier to exert effects within the central nervous system. Their interaction with gut-brain pathways may directly or indirectly influence brain function, learning, memory, and mood^8^.

In individuals with AN, levels of butyrate-producing bacteria and fecal butyrate are often reduced, likely due to decreased dietary fiber intake resulting from restrictive and pathological eating habits. Butyrate has been associated with improved metabolic outcomes and reduced levels of anxiety and depression, suggesting a potential link between microbial changes and psychiatric symptoms in AN^9^.

Beyond SCFAs, the gut microbiota also modulates neurotransmitter production and can independently produce several neurotransmitters. For example, serotonin (5-HT), synthesized from the essential amino acid tryptophan – abundant in protein-rich foods such as chicken, eggs, and dairy – plays critical roles in gastrointestinal motility and mood regulation. A disrupted microbial environment may impair these functions by altering 5-HT signaling.

As with many other diseases, there is an intensive search for therapeutic interventions that can restore the gut microbiota to a “healthy” state and support achieving and maintaining “healthy” eating behaviors. Given the role of the gut microbiota in regulating appetite, mood, and metabolism, its restoration in individuals with AN is considered a stepping stone toward a more complete recovery. Although partial microbiome recovery may occur during inpatient treatment and weight restoration, dysbiosis often persists even as body mass index (BMI) and psychological symptoms improve^6^. This raises concerns about the adequacy of microbiome normalization in AN, particularly in chronic cases or those that are treatment-resistant. Understanding and targeting gut dysbiosis may be essential for promoting long-term recovery and developing microbiota-based interventions.

This study aimed to characterize gut microbiota composition and metabolomic profiles in patients with early-stage (acute) AN and SEAN, and to identify factors associated with disease persistence. We observed distinct differences in gut microbiota composition, diversity, and microbial metabolites among acute AN, SEAN, and healthy controls. Notably, markers of increased intestinal permeability and low-grade systemic inflammation were more pronounced in SEAN than in acute AN, suggesting progressive deterioration of gut barrier function over time. This study addresses a critical gap in the literature by differentiating microbiome alterations across stages of AN. Elucidating the mechanisms underlying the transition from acute to chronic AN may inform novel preventive and therapeutic strategies targeting the gut microbiota.

## Results

### Clinical, anthropometric, and biochemical description of the study cohort

The study included 62 patients with AN (acute AN = 29; SEAN = 33) and 30 healthy women, i.e., healthy controls (HCs) (Table 1). Patients with acute AN were younger than the other participants, reflecting a typical age of disease onset of between 12 and 25 years^10^. As expected, disease duration differed between the acute AN and SEAN groups (Table 1). Both AN groups exhibited significant differences from healthy controls in the malnutrition-related physical measures, including body weight, BMI, body fat percentage, and waist and hip circumference. Additionally, hyperactivity, commonly observed in AN, was also more prevalent in the AN groups vs. HCs. AN patients also used more antidepressants, antipsychotics, and anxiolytics than the HCs. SEAN patients reported greater adult-life stress and higher antidepressant use compared to the acute AN patients (Table 1). Although comorbidities were common among AN patients, their prevalence did not differ significantly between the two groups (Table S1).

**Table 1:** Clinical, anthropometric, and biochemical parameters, and the medication used in the study cohort

Variable	Controls (HC) (*n* = 30)	Acute AN (*n* = 29)	SEAN (*n* = 33)	*p*-values		
				HC vs. Acute AN	HC vs. SEAN	Acute AN vs. SEAN
**Clinical parameters**						
Age (years)	26 (23.8; 30)	20 (18; 26)	27 (21.5; 31)	0.003**	NS	0.0014**
Disease duration (months)	–	24 (15; 35)	102 (84; 150)	–	–	<0.0001****
Severity of AN (DSM)	–	3 (2; 4)	4 (2.5; 4)	–	–	NS
AN type (R/P)	–	18/11	21/12	–	–	NS
Childhood stress (%)	3.3	10.3	6.1	NS	NS	NS
Adolescent stress (%)	30	27.6	51.5	NS	NS	NS
Adulthood stress (%)	20	13.8	51.5	NS	0.0175*	0.004**
Hyperactivity	0 (0; 0)	1 (0; 2)	0 (0; 2)	<0.0001****	0.007***	NS
Allergy (%)	33.4	48.3	45.5	NS	NS	NS
**Anthropometric and biochemical parameters**						
Height (cm)	169.6 ± 6.89	167.00 ± 5.27	164.60 ± 6.73	NS	NS	NS
Weight (kg)	64.34 ± 7.29	43.48 ± 4.32	38.32 ± 6.97	<0.0001****	<0.0001****	NS
BMI (kg/m^2^)	22.35 ± 2.00	15.60 ± 1.33	14.14 ± 2.31	<0.0001****	<0.0001****	NS
Body Fat (%)	27.73 ± 5.03	6.23 ± 4.28	5.52 ± 4.54	<0.0001****	<0.0001****	NS
Waistline (cm)	70.70 ± 6.20	61.10 ± 4.29	59.15 ± 8.67	0.0395*	<0.0041**	NS
Hipline (cm)	95.70 ± 5.31	80.10 ± 4.07	76.42 ± 5.55	<0.0059**	<0.0002***	NS
TAG (mmol/l)	0.90 ± 0.33	0.88 ± 0.34	1.09 ± 0.97	NS	NS	NS
Cholinesterase (ukat/l)	118.50 ± 24.47	96.10 ± 25.90	110.10 ± 48.14	<0.0003****	0.0315***	NS
Albumin (g/l)	47.17 ± 2.96	46.51 ± 3.24	45.63 ± 3.45	NS	NS	NS
IgG (g/l)	12.01 ± 2.14	10.61 ± 1.89	10.19 ± 1.70	NS	NS	NS
IgA (g/l)	2.06 ± 0.79	1.76 ± 0.64	2.21 ± 0.53	NS	NS	0.0062**
IgM (g/l)	1.58 ± 0.74	1.57 ± 0.84	1.28 ± 0.63	NS	0.0354*	0.0474*
TSH (mIU/L)	2.68 ± 1.28	2.41 ± 1.25	2.74 ± 1.56	NS	NS	NS
fT4 (pmol/l)	14.65 ± 1.35	12.84 ± 1.73	12.84 ± 2.40	NS	NS	NS
fT3 (pmol/l)	5.54 ± 0.50	3.88 ± 1.04	4.04 ± 0.91	<0.0001****	<0.0001****	NS
**Used medications**						
Antidepressants (%)	0	65.5	90.9	<0.0001****	<0.0001****	0.0263*
Antipsychotics (%)	0	31.0	48.5	0.0008***	<0.0001****	NS
Anxiolytics (%)	0	24.1	15.2	0.0046**	0.054*	NS
Contraceptives (%)	16.7	10.3	24.3	NS	NS	NS
Thyroid hormones (%)	6.7	17.2	30.0	NS	NS	NS

The comparison of age between groups was evaluated by the Kruskal-Wallis test with Dunn´s multiple comparison test. Comparison between the 2 groups of AN patients was evaluated using the Mann-Whitney test. Categorical data were evaluated using Fisher’s exact test. Multiple comparisons of anthropometric and biochemic data between groups were evaluated using 2-way ANOVA with Tukey corrections on transformed data (λ = 0.1). The results are shown as median with IQR (age, disease duration, severity of AN, hyperactivity), proportional numbers (AN type), percentage (stress, allergy, medication usage), or mean ± SD (anthropometric and biochemical parameters).

*NS* non-significant, *SEAN* severe and enduring AN, *TAG* triacylglyceride, *TSH* thyroid-stimulating hormone, *fT4* free thyroxine, *fT3* free triiodothyronine.

Stars indicate *p*-values for each comparison. *p < 0.05; **p < 0.01; ***p < 0.001; ****p < 0.0001.

Statistically significant values are shown in bold.

**Fig. 1:**
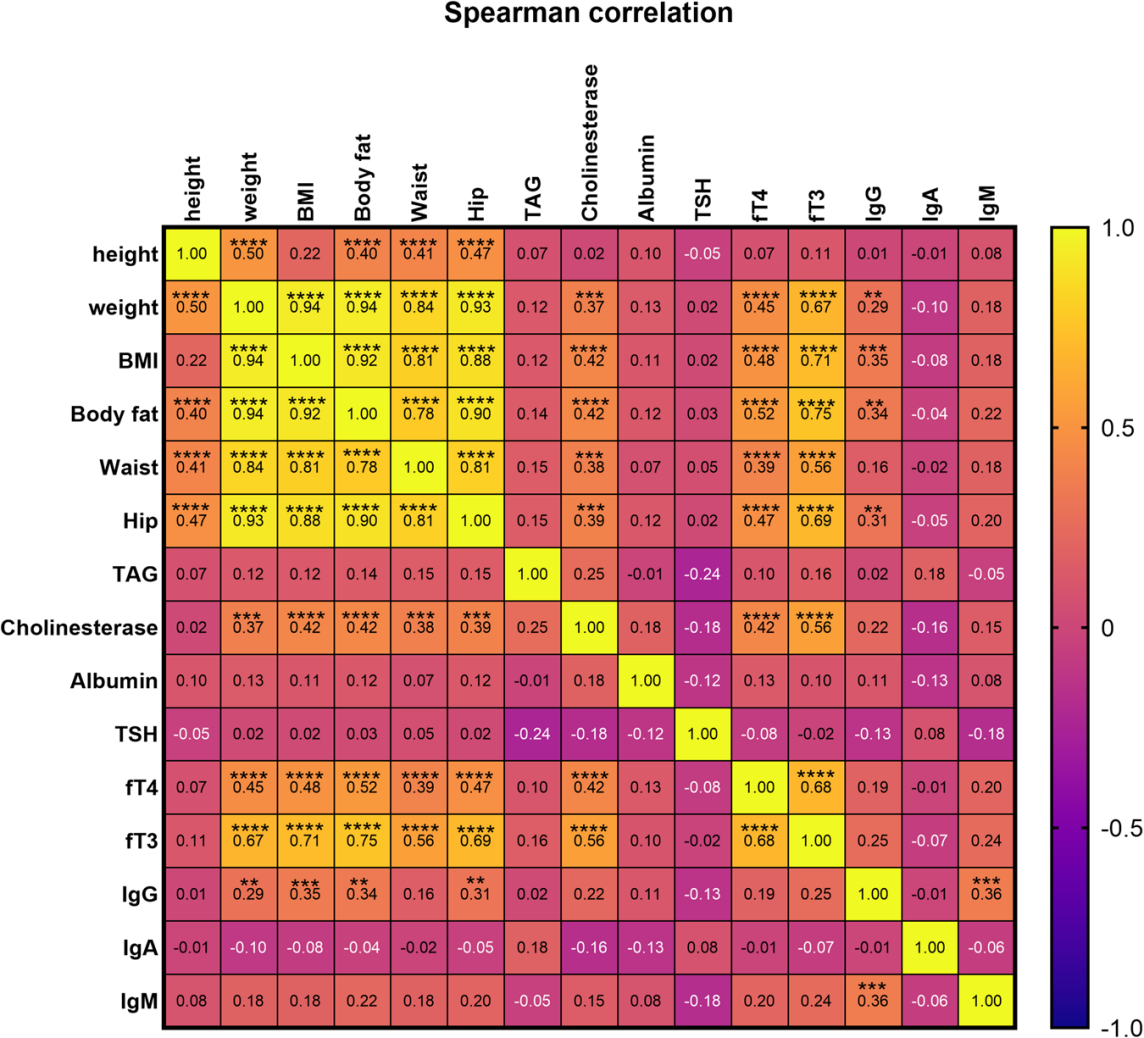
Correlation matrix between anthropometric and biochemical parameters. The heatmap matrix displays Spearman correlation coefficients between parameters for all patients and healthy controls. Yellow indicates a positive correlation, while blue represents a negative correlation. Asterisks denote the *p*-values for each comparison: **p < 0.01, ***p < 0.001, ***p < 0.0001. Correlations with *p*-values ≥ 0.01 are not shown. BMI body mass index, TAG triacylglycerides, TSH thyroid-stimulating hormone, fT4 free thyroxine, fT3 free triiodothyronine.

Biochemically, both AN groups had reduced serum cholinesterase and free triiodothyronine (fT3) levels. Spearman correlation analysis (Fig. 1) revealed that anthropometric measures of body size were positively correlated with serum cholinesterase, free thyroid hormones (fT3 and ft4), and IgG levels. SEAN and acute AN patients also differed in IgA and IgM levels, possibly reflecting some measure of gastrointestinal (GI) damage (Table 1).

### Patients with acute AN had a higher EDE-Q and HAMD total score

**Fig. 2:**
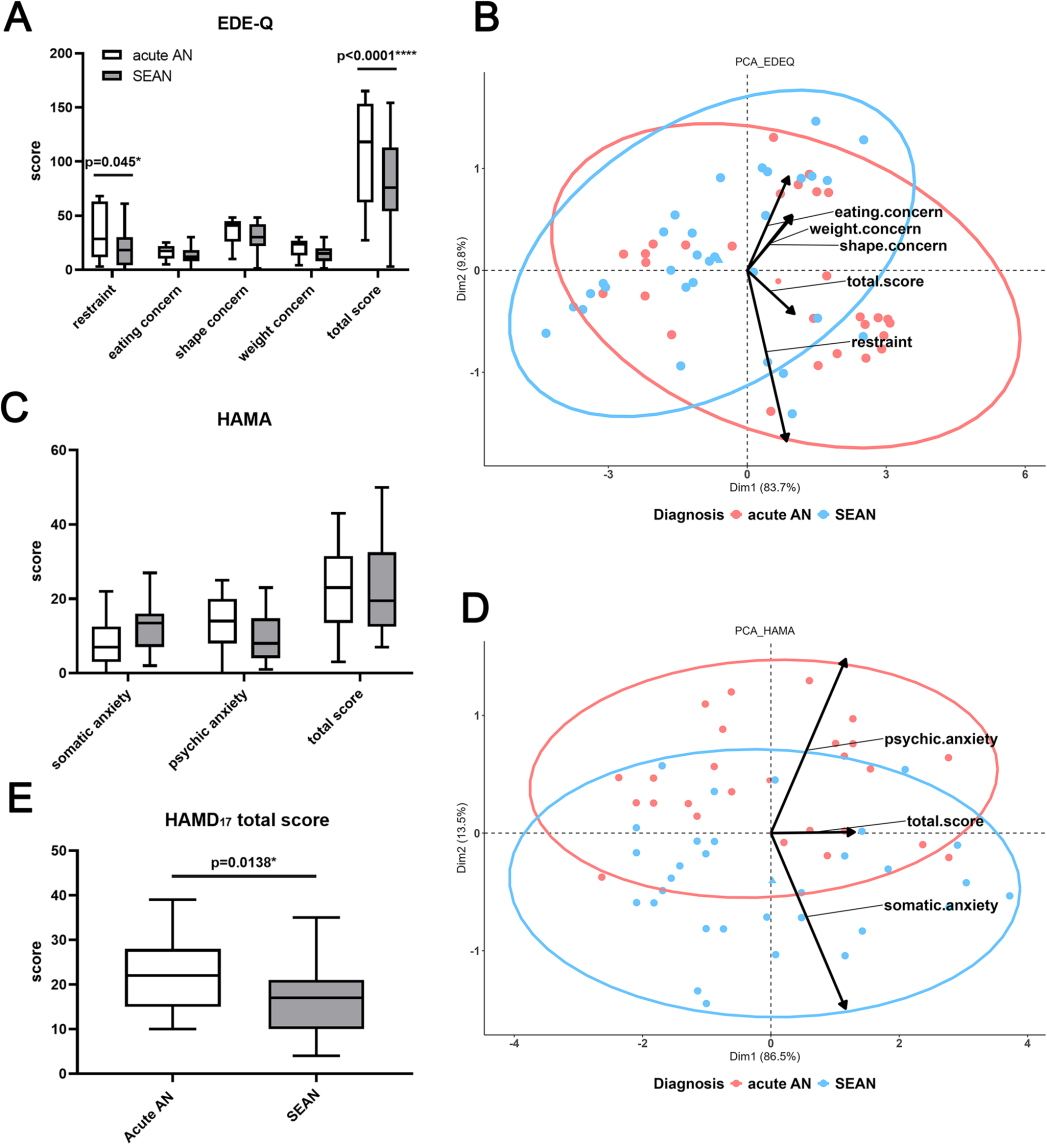
Comparison of subscale and total scores, along with principal component analyses (PCA), of the Eating Disorder Examination Questionnaire (EDE-Q), Hamilton Anxiety Rating Scale (HAMA), and Hamilton Depression Rating Scale (HAMD) in patients with acute anorexia nervosa (AN) and severe and enduring AN (SEAN). **A** Subscale and total scores from the Eating Disorder Examination Questionnaire (EDE-Q); **B** Principal component analysis (PCA) of EDE-Q subscale and total scores; **C** Subscale and total scores from the Hamilton Depression Rating Scale (HAMD); **D** PCA of HAMD subscale and total scores; **E** Total scores from the HAMD_17_. Data were transformed using the Box-Cox transformation (λ = 0.1) and analyzed using two-way ANOVA with Tukey’s multiple comparison test. Asterisks indicate statistical significance: *p < 0.05, ****p < 0.0001.

To assess psychiatric differences between acute AN and SEAN, patients completed the Eating Disorder Examination Questionnaire (EDE-Q), the Hamilton Anxiety Rating Scale (HAMA), and the Hamilton Depression Rating Scale (HAMD). SEAN patients exhibited lower total EDE-Q scores and dietary restraint subscores (reflecting extreme attempts to limit food intake) compared to those with acute AN, which showed less restrictive eating behavior (Fig. 2A, B). Although HAMA scores did not differ significantly between groups, principal component analysis (PCA) revealed a trend toward psychological anxiety in acute AN and somatic anxiety in SEAN (Fig. 2C, D). Acute AN patients also had higher HAMD scores, likely reflecting their lower use of antidepressants relative to SEAN patients (Fig. 2E).

### I-FABP levels are elevated in patients with SEAN and correlate with BMI

**Fig. 3:**
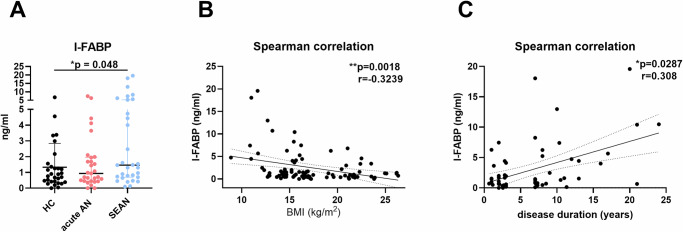
Levels of intestinal fatty acid-binding protein (I-FABP) in the serum of healthy controls and patients with acute AN or SEAN and Spearman correlation. **A** Serum levels of intestinal fatty acid binding protein (I-FABP); **B** Spearman correlation between I-FABP values and body mass index (BMI), with 95% confidence intervals, in patients with anorexia nervosa (AN) and healthy controls (HC); **C** Spearman correlation between I-FABP values and disease duration in patients with AN, with 95% confidence intervals. Data were transformed using the Box-Cox transformation (λ = 0.1) and analyzed using one-way ANOVA with Tukey’s multiple comparison test. Asterisks indicate statistical significance: *p < 0.05, **p < 0.01. Boxplots represent medians with interquartile ranges. HC healthy controls, SEAN severe and enduring AN.

To evaluate potential damage to the intestinal mucosa, we measured serum levels of intestinal fatty acid binding protein (I-FABP), a marker released upon epithelial injury. Patients with SEAN exhibited moderately elevated I-FABP levels, driven by a subset of SEAN patients with particularly high values (Fig. 3A). Spearman correlation analysis across all participants revealed a negative correlation between I-FABP and BMI, and a positive correlation with disease duration in AN patients (Fig. 3B, C), suggesting greater GI mucosal damage associated with chronic illness and lower body weight.

### Patients with anorexia have different levels of inflammatory and intestinal barrier function markers

**Fig. 4:**
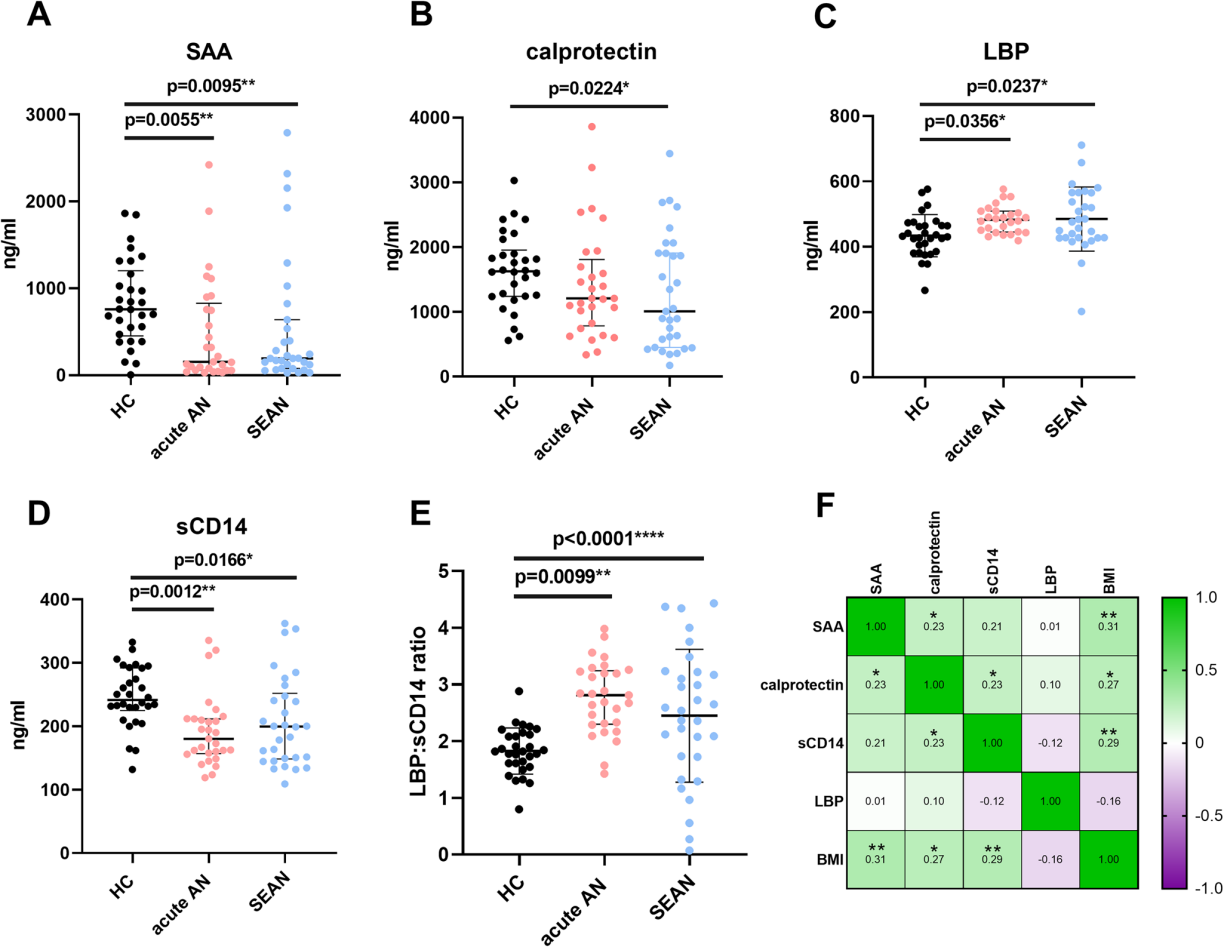
Serum levels of inflammatory and intestinal barrier function markers. **A** Serum levels of serum amyloid A (SAA); **B** Levels of lipopolysaccharide-binding protein (LBP); **C** Levels of secretory CD14 (sCD14); **D** Serum calprotectin levels; **E** LBP:sCD14 ratio; **F** Spearman correlation coefficients between serum parameters and body mass index (BMI). Green indicates a positive correlation; purple indicates a negative correlation. Data were transformed using the Box-Cox transformation (λ = 0.1) and analyzed using one-way ANOVA with Tukey’s multiple comparison test. Asterisks denote statistical significance: *p < 0.05; **p < 0.01; ***p < 0.001; ****p < 0.0001. Boxplots show medians with interquartile ranges. Sample sizes: HC = 30, acute AN = 29, SEAN = 33. SAA serum amyloid A, LBP lipopolysaccharide-binding protein, sCD14 secretory CD14, BMI body mass index.

To determine whether AN is associated with inflammation, we measured serum levels of the inflammatory biomarkers serum amyloid A (SAA) and calprotectin in all participants. Surprisingly, both biomarkers were reduced in AN patients compared to HCs, with calprotectin levels significantly lower in SEAN (Fig. 4A, B).

To investigate whether AN is associated with increased endotoxin exposure, we measured serum levels of two putative biomarkers of GI barrier function: lipopolysaccharide-binding protein (LBP) and secretory CD14 (sCD14). Both AN groups exhibited lower levels of sCD14, while LBP was slightly more elevated in SEAN (Fig. 4D, C, respectively). These differences were further accentuated in the LBP:sCD14 ratio, with both patient groups displaying higher serum ratios compared to healthy controls (Fig. 4E). Spearman correlation analyses revealed positive correlations between serum amyloid A (SAA), calprotectin, and sCD14 levels with BMI, whereas LBP levels showed no such relationship (Fig. 4F). To assess whether the observed differences in microbial translocation markers were independent of BMI, we performed ANOVA models including disease status and BMI as predictors, using Box-Cox transformed levels of sCD14, SAA, and calprotectin as dependent variables. Disease status remained a significant predictor of sCD14 (*p* = 0.007) and SAA levels (*p* = 0.004), whereas BMI had no significant association (*p* = 0.969 and *p* = 0.741, respectively). For calprotectin, disease status approached significance (*p* = 0.07), while BMI again showed no significant association (*p* = 0.307).

### Gut microbiota composition differs in both acute AN and SEAN

**Fig. 5:**
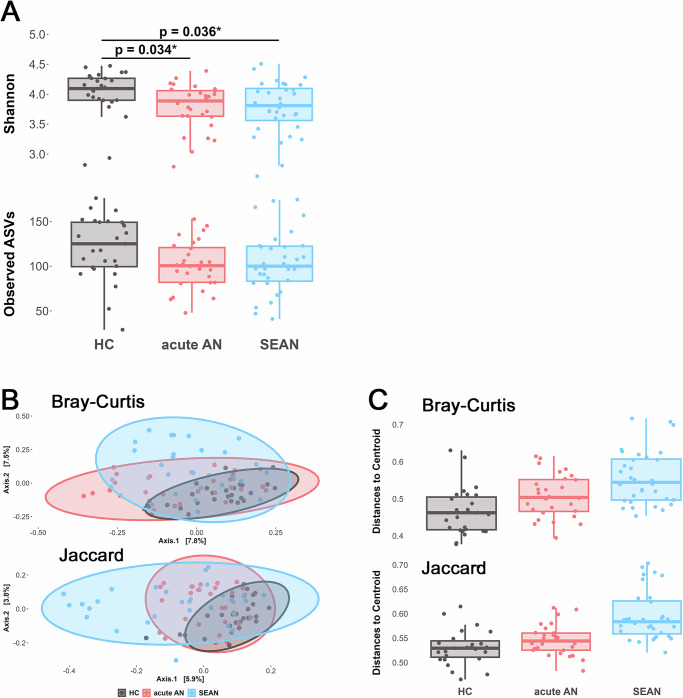
Variation in alpha diversity, composition, and dispersion of gut microbiota among studied groups. **A** Boxplots show variation in gut microbiota alpha diversity, measured by Shannon diversity and ASV richness, across groups. Boxplots represent median values with interquartile ranges. **B** Principal coordinate analysis (PCoA) of Bray-Curtis and Jaccard dissimilarities reveals differences in gut microbiota composition between groups. Bray-Curtis PCoA plots show that the x-axis explains 7.8% and the y-axis 7.5% of variation; Jaccard plots show 5.9% and 3.8%, respectively. Black dots represent healthy controls (HC), red dots indicate acute AN patients, and blue dots represent SEAN patients. Shaded ellipses denote 90% confidence intervals illustrating inter-individual variation. **C** Multivariate analysis of microbial dispersion based on Bray-Curtis and Jaccard dissimilarities. Individual points in boxplots represent distances to the centroid in multivariate space for each group. AN anorexia nervosa, HC healthy controls, SEAN severe and enduring AN.

Both acute AN and SEAN patients exhibited lower alpha diversity compared to healthy controls, based on the Shannon Index (ANOVA: *p* = 0.018, F = 4.423; Tukey post hoc test: *p* = 0.034 for HC vs. acute AN and *p* = 0.036 for HC vs. SEAN; Fig. 5A). A similar trend was observed for Amplicon Sequence Variant (ASV) richness, although this difference was marginally non-significant (ANOVA: *p* = 0.051, F = 3.09; Fig. 5A).

Microbial community composition also differed significantly among the three (HC, AN, and SEAN) groups based on Bray-Curtis and Jaccard dissimilarities (Fig. 5B and Table 2), including differences between acute AN and SEAN. These group-level differences remained significant even after adjusting for BMI, body fat percentage, and presence of vomiting (PERMANOVA: Bray-Curtis: *p* = 0.002, F = 1.464; Jaccard: *p* = 0.004, F = 1.269).

**Table 2:** Comparisons of interindividual variation and composition of gut microbiota

	Bray-Curtis				Jaccard		
PERMANOVA	df	F	p	R^2^	F	p	R^2^
HC vs. acute AN	2;84	2.003	0.002**	0.0364	1.58	0.001***	0.0289
HC vs. SEAN	2;84	2.797	0.001***	0.0468	2.256	0.001***	0.0381
acute AN vs. SEAN	2;84	1.832	0.003**	0.0306	1.175	0.002**	0.0252

Betadisper	df	F	p		F	p	
HC vs. acute AN	2;84	14.607	0.0467*		15.824	0.676	
HC vs. SEAN	2;84		<0.0001****			<0.0001****	
acute AN vs. SEAN	2;84		0.0124*			<0.0001****	

Comparisons of (A) microbial composition and (B) interindividual variation between studied groups based on PERMANOVA and Betadisper tests, respectively. Tests were conducted using relative abundance-based (Bray–Curtis) and prevalence-based (Jaccard) dissimilarities. Values of (pseudo-) F statistics (F), associated degrees of freedom (df), resulting probability values (p), and proportions of explained variance (R2) are shown. Significant values are in bold type.

Stars indicate p-values for each comparison. *p < 0.05, **p < 0.01, ***p < 0.001, ****p < 0.0001.

Betadisper analysis indicated a significant increase in interindividual variability in gastrointestinal microbiota composition among SEAN patients, relative to both healthy controls (HCs) and individuals with acute AN, as assessed using Bray-Curtis and Jaccard dissimilarity metrics (Fig. 5C and Table 2). Patients with acute AN also demonstrated greater variability than HCs, but only relative to the Bray-Curtis dissimilarity (Table 2).

**Fig. 6:**
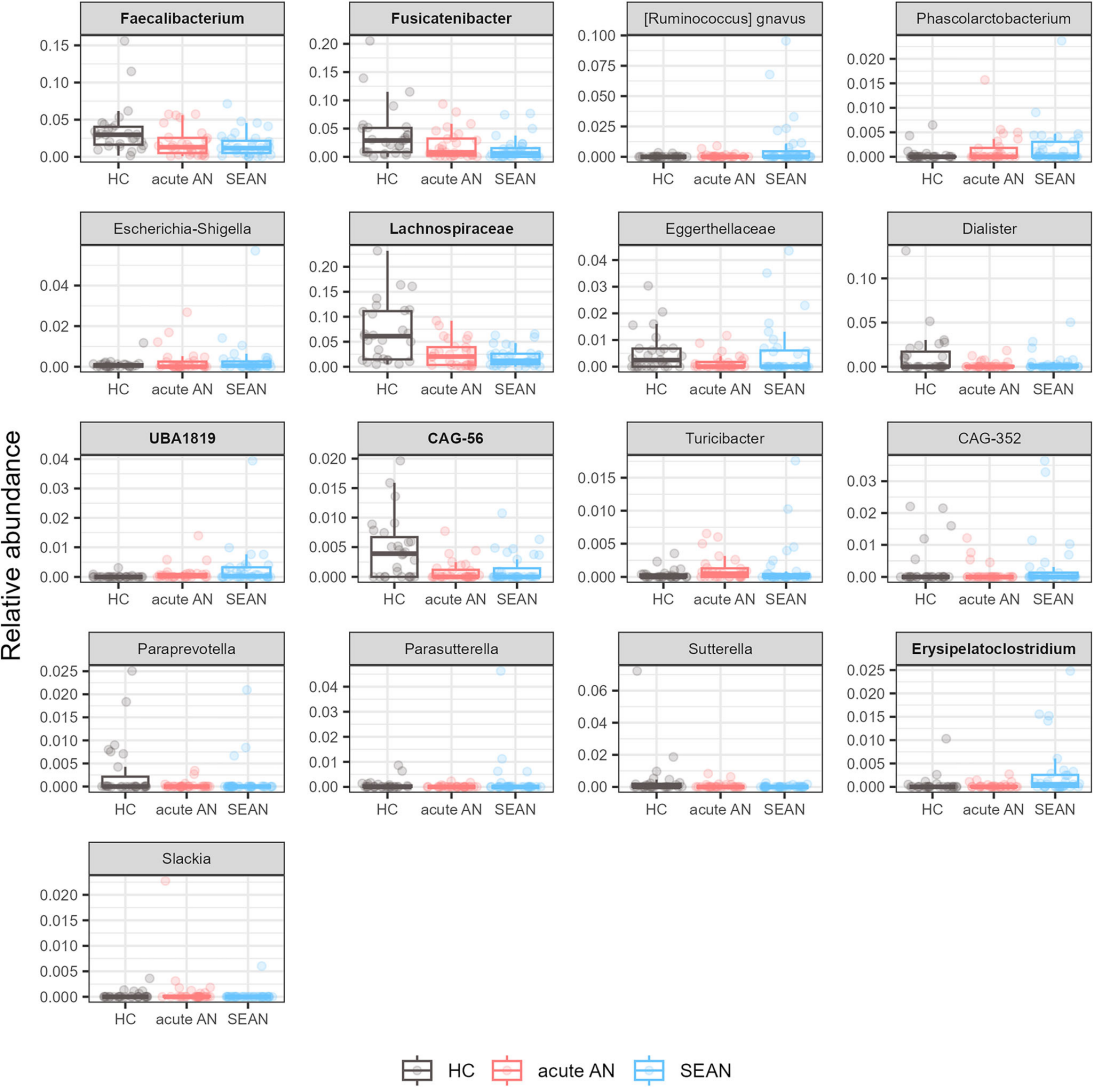
Differential relative abundance analysis at the genus level. Boxplots display bacterial genera with significantly different relative abundance across study groups, as identified by ANCOM-BC2 (FDR-adjusted q < 0.05, Holm correction). Genera that passed sensitivity filtering are shown in bold.

Despite overall similarities in the relative abundance of dominant bacterial classes and genera (Fig. S1), differential abundance testing using ANCOM-BC2 identified 17 bacterial taxa with significantly altered distributions across groups. Of these, six taxa passed sensitivity filtering: *Erysipelatoclostridium*, *CAG-56*, *Fusicatenibacter*, *Faecalibacterium*, *UBA1819*, and *Lachnospiraceae* (Fig. 6). Post-hoc analysis (Fig. S2) revealed that (1) *UBA1819* was reduced, and *Lachnospiraceae* increased in both AN groups compared to HCs, (2) *Erysipelatoclostridium* was enriched in SEAN relative to acute AN, (3) *CAG-56* was reduced in acute AN compared to HCs.

Significant associations (*p* < 0.05) were observed between alpha diversity (Shannon Index) and several clinical variables, including BMI, body fat percentage, vomiting, and the use of antidepressants and antipsychotics (Table 3). No significant associations were found between Shannon diversity and AN subtype (restrictive vs. purgative), AN severity, i.e., based on the Diagnostic and Statistical Manual of Mental Disorders (DSM), hyperactivity, stress, psychiatric or physical comorbidities, hypothyroidism, malnutrition, psychometric scores (EDEQ, HAMA, HAMD), lipopolysaccharide-binding protein (LBP) levels, or other biochemical parameters.

**Table 3:** Alpha and beta diversity associations

Alpha diversity (Shannon index)				
	F-value		*p*-value	
BMI	5.149		**0.0258**	
Body fat %	5.127		**0.0261**	
Vomiting	6.091		**0.0156**	
Antidepressants	5.700		**0.0192**	
Antipsychotics	5.813		**0.0181**	

Beta diversity				
	Bray-Curtis		Jaccard	
	F-value	*p*-value	F-value	*p*-value
Body fat %	2.092	**0.001**	1.643	**0.002**
AN type (res/purg)	1.405	**0.031**	NS	NS
Vomiting	1.555	**0.015**	1.283	**0.035**
Antidepressants	1.743	**0.005**	1.446	**0.005**
Hormonal contraception	1.553	**0.020**	1.314	**0.030**
Stress	1.596	**0.010**	1.327	**0.015**
Other psychiatric disorders	1.820	**0.005**	1.467	**0.005**
OCD	1.586	**0.015**	1.301	**0.010**
fT3	2.388	**0.005**	1.791	**0.005**

*AN type* restrictive (res) or purgative (purg), *BMI* body mass index, *fT3* free triiodothyronine, *OCD* obsessive-compulsive disorder. Statistically significantvalues are shown in bold.

Beta diversity showed significant associations (*p* < 0.05) with body fat, AN subtype, psychiatric comorbidity (including obsessive-compulsive disorder), stress, vomiting, use of antidepressants and hormonal contraceptives, and free triiodothyronine (fT3) levels (Table 3).

No associations were detected between beta diversity and AN severity, hyperactivity, hypothyroidism, malnutrition, psychometric scores, LBP levels, or other biochemical markers.

### Predictive functional profiling of the gut microbiota

We used the PICRUSt2 pipeline to infer potential functional capacities of microbial communities based on 16S rRNA gene profiles. Predicted enzyme functions differed significantly between the analyzed groups (PERMANOVA, Bray-Curtis: *p* = 0.046, F = 2.684), with ANCOM-BC2 analyses revealing significant differences in the abundance of predicted functions (Fig. S3 and Table S2). The most pronounced disparities were observed between HCs and SEAN patients. The most affected predicted enzyme functions were related to amino acid metabolism, detoxification, oxidative stress response, and carbohydrate metabolism. These results represent inferred functional potential rather than direct measurements of microbial metabolic activity and should therefore be interpreted with caution.

### Fecal levels of neurotransmitters, tryptophan, and SCFAs differed between AN patients and healthy controls

**Fig. 7:**
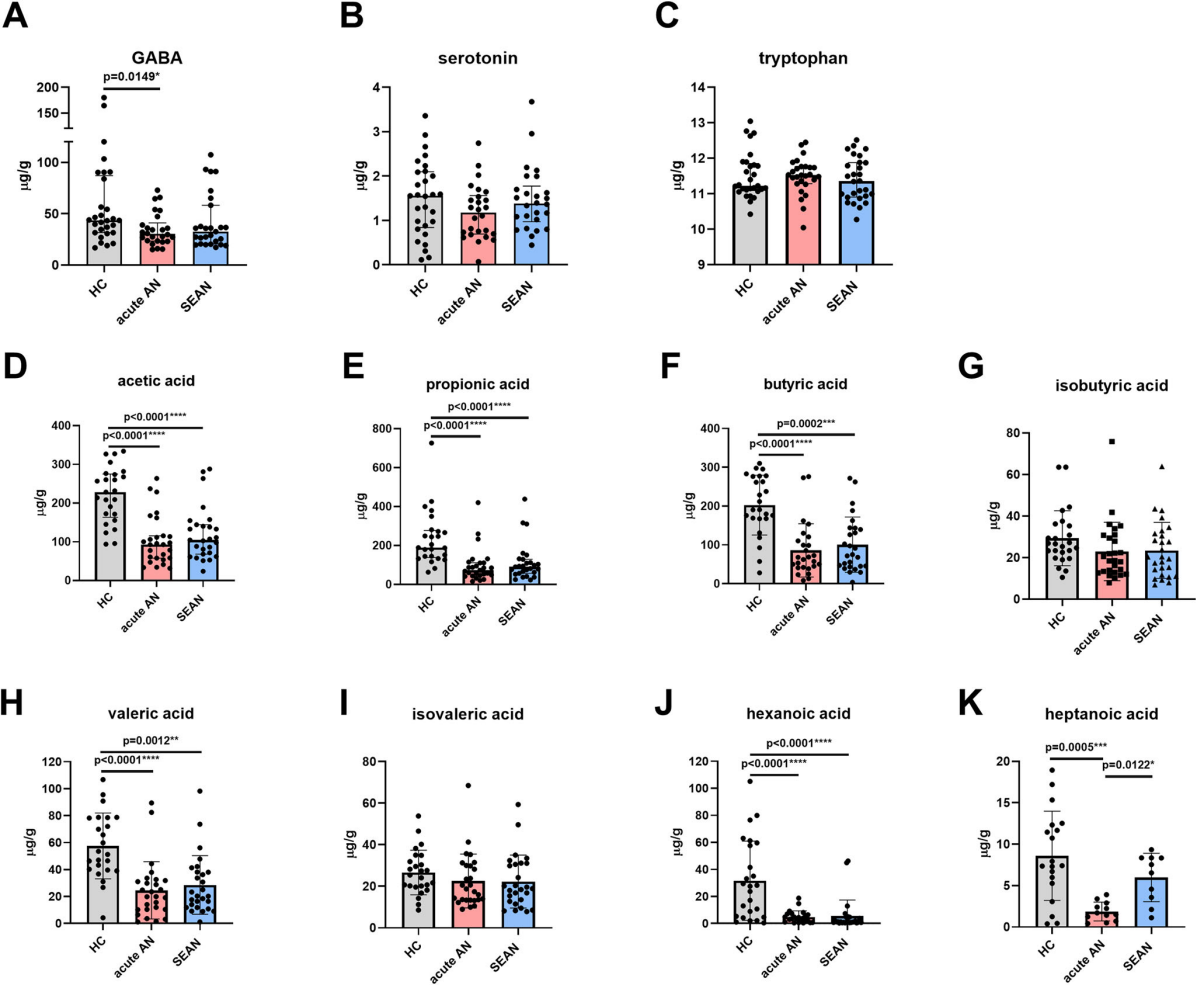
Concentrations of fecal neurotransmitters, serotonin precursors, and short-chain fatty acids (SCFAs) in the three studied groups. Relative concentrations of selected fecal metabolites and short-chain fatty acids (SCFAs) across study groups: **A** GABA, **B** serotonin, **C** tryptophan, **D** acetic acid, **E** propionic acid, **F** butyric acid, **G** isobutyric acid, **H** valeric acid, **I** isovaleric acid, **J** hexanoic acid, **K** heptanoic acid. Data were Box-Cox transformed (λ = 0.1) and analyzed using one-way ANOVA with Tukey’s multiple comparisons test. Asterisks denote significance thresholds: *p* < 0.05 (*), < 0.01 (**), < 0.001 (***), < 0.0001 (****). Boxplots represent medians with interquartile ranges. AN anorexia nervosa, GABA gamma-aminobutyric acid, HC healthy controls, SEAN severe and enduring AN.

Fecal concentrations of neurotransmitters, their precursors, and SCFAs were quantified using liquid chromatography in HCs and AN patients. GABA levels were slightly higher in HCs compared to AN patients, while serotonin and its precursor tryptophan showed no significant group differences (Fig. 7). Kynurenine, a tryptophan metabolite, was detected in approximately 25% of samples and excluded from further analysis. Dopamine and hydroxytryptophan concentrations were below the quantification threshold in all samples.

Gas chromatography analysis revealed significant differences in fecal SCFA concentrations between HCs and AN patients. Healthy controls exhibited significantly higher levels of acetic acid, propionic acid, butyric acid, valeric acid, and hexanoic acid compared to either AN group. No significant differences were found for isobutyric and isovaleric acids. Heptanoic acid concentrations were significantly elevated in HCs compared to acute AN patients and represented the only SCFA present at higher levels in SEAN than in acute AN, although the difference was modest (Fig. 7). Formic acid and 2-methylvaleric acid were excluded from analysis due to low detection rates across samples.

Valeric, butyric, acetic, and propionic acid concentrations were strongly intercorrelated (Fig. S4), as were isovaleric and isobutyric acids, though with slightly lower strengths (ρ < 0.8). GABA showed moderate correlations with valeric, butyric, acetic, and propionic acids. Tryptophan was significantly correlated only with propionic acid, while serotonin exhibited no significant correlations with SCFAs or other neurotransmitters.

### Serum levels of neurotransmitters, tryptophan, and SCFAs differ between AN patients and healthy controls

**Fig. 8:**
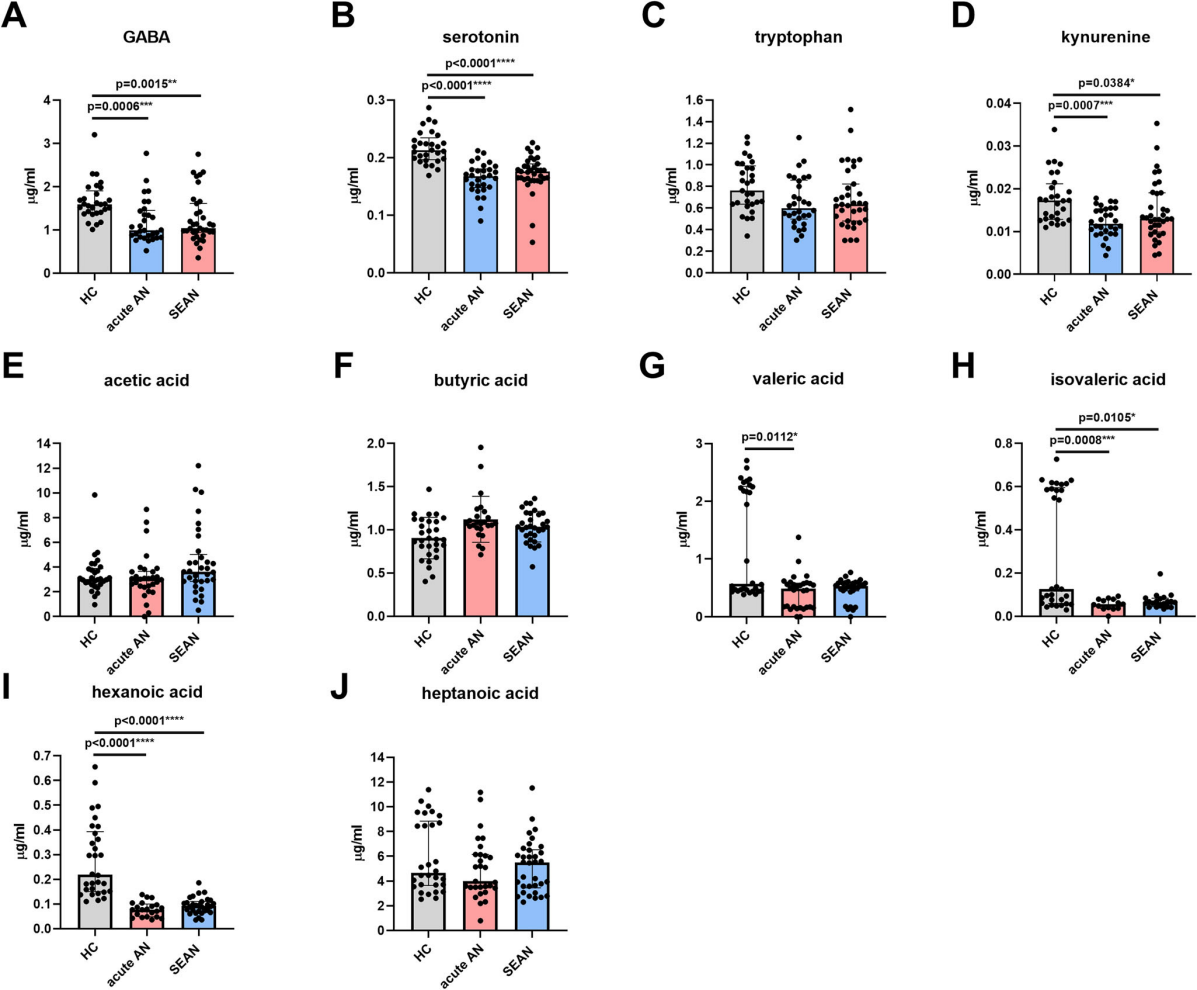
Concentrations of serum neurotransmitters, serotonin precursors, and short-chain fatty acids (SCFAs) in the three studied groups. **A** GABA; **B** serotonin; **C** tryptophan; **D** kynurenine ; **E** acetic acid; **F** butyric acid; **G** valeric acid; **H** isovaleric acid; **I** hexanoic acid; **J** heptanoic acid. Data were transformed using the Box-Cox transformation (λ = 0.1) and analyzed using a one-way ANOVA with Tukey’s multiple comparison test. Asterisks indicate *p*-values for each comparison. *p < 0.05; **p < 0.01; ***p < 0.001; ****p < 0.0001. Boxplots show medians with interquartile ranges. AN anorexia nervosa, GABA gamma-aminobutyric acid, HC healthy controls, SEAN severe and enduring AN.

Serum concentrations of neurotransmitters, tryptophan, and SCFAs were measured in AN patients and HCs. Overall, serum levels of neurotransmitters and SCFAs per milliliter were lower than their corresponding concentrations per gram of stool. GABA, serotonin, and kynurenine levels were significantly reduced in both AN groups compared to HCs, while tryptophan levels did not differ between AN subtypes and mirrored those observed in fecal samples. Dopamine and hydroxytryptophan concentrations were below the quantification limit in all serum samples (Fig. 8).

Serum concentrations of hexanoic acid, valeric acid, and isovaleric acid were significantly reduced in AN patients compared to HCs (Fig. 8). Unlike stool samples, propionic acid and isobutyric acid were detectable in only a minority of serum samples and were excluded from further analysis.

### GABA, serotonin, and tryptophan correlated with several bacterial taxa

**Fig. 9:**
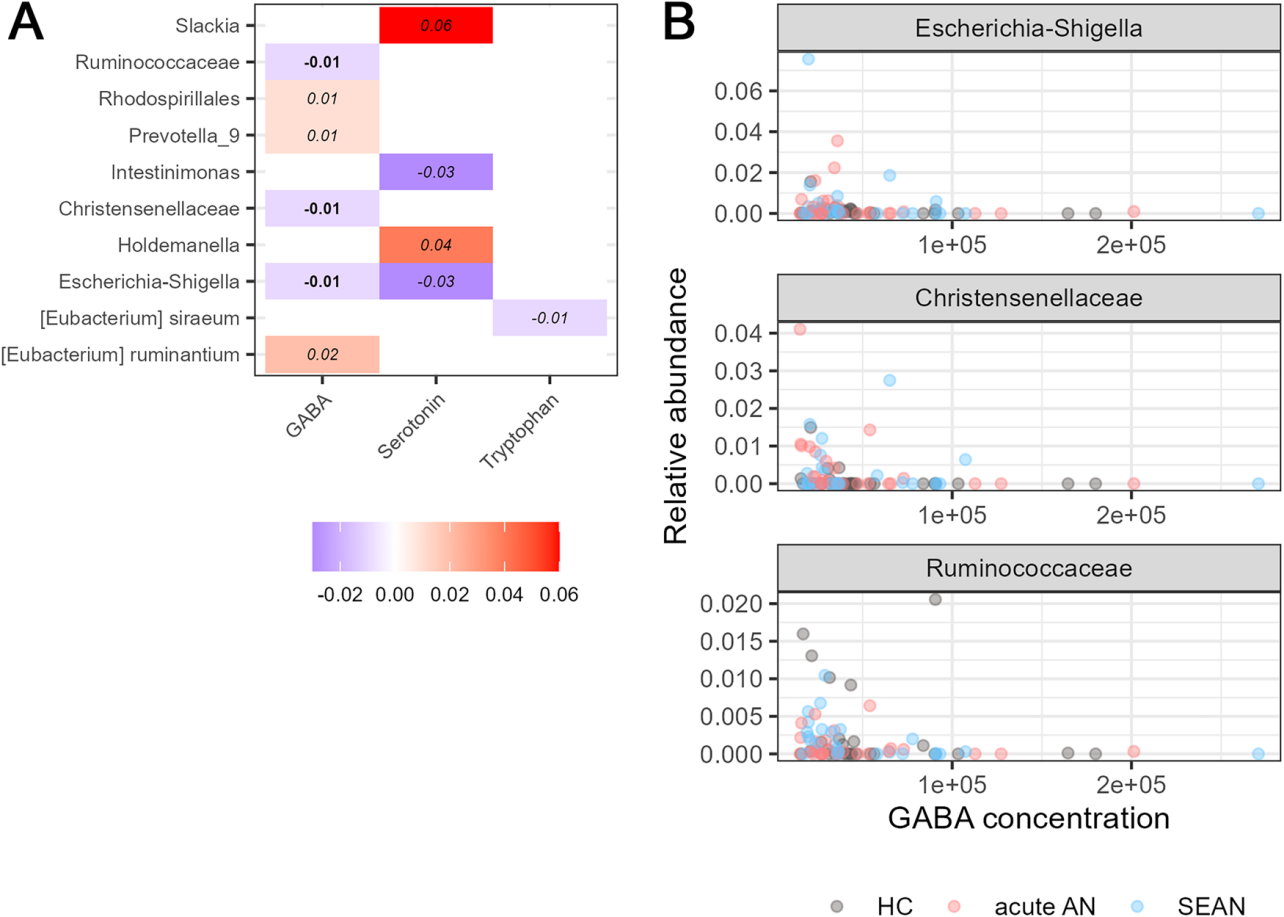
Associations between GABA concentrations and the bacterial taxa. **A** The log-fold changes are indicated by a color scale and numerical values within the cells. The color is white if the corresponding pairwise test was not significant. Numbers are in italics if the coefficient did not pass the sensitivity test in the differential abundance analysis; otherwise, they are in bold. **B** Significant associations between GABA concentrations and the relative abundance of bacterial taxa that passed the ANCOM-BC2 sensitivity test. (FDR-adjusted q < 0.05 Holm correction).

We identified significant associations between the relative abundance of specific bacterial taxa and the concentrations of GABA, serotonin, and tryptophan (Fig. 9). GABA was negatively correlated with *Christensenellaceae*, *Ruminococcaceae*, and *Escherichia-Shigella*, all of which remained significant following ANCOM-BC2 sensitivity testing. Two taxa were associated with serotonin levels, and one was linked to tryptophan, though only the GABA-related associations passed the sensitivity threshold (Fig. 9).

**Fig. 10:**
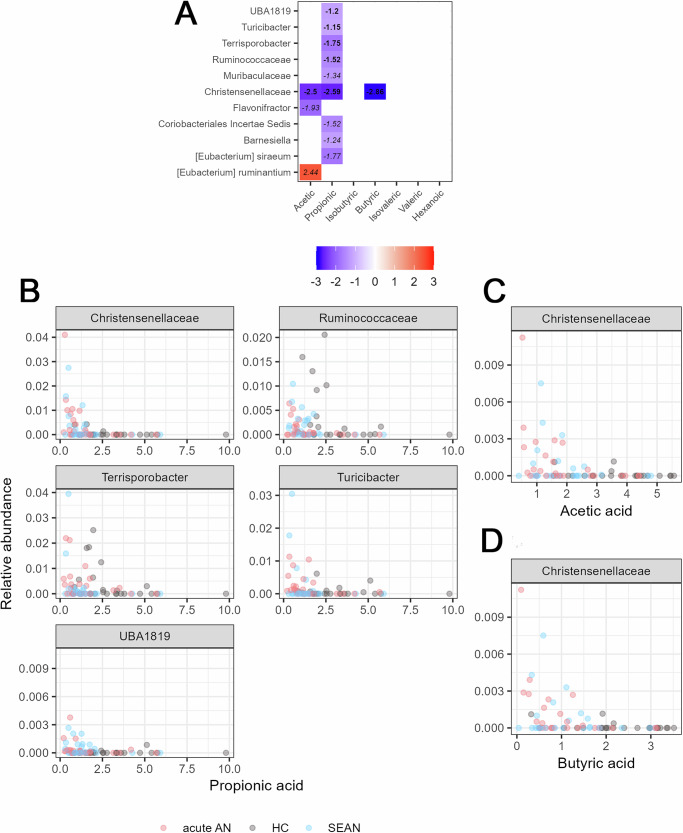
Associations between SCFA concentrations and bacterial taxa. **A** Log-fold changes are represented by a color scale and numeric values within each cell. Cells are white if the corresponding pairwise test was not significant. Italicized values indicate coefficients that did not pass the sensitivity test in the differential abundance analysis; bold values passed the sensitivity threshold. **B**–**D** Show significant associations between SCFA concentrations—**B** propionic acid, **C** acetic acid, and **D** butyric acid—and the relative abundance of bacterial taxa that passed ANCOM-BC2 sensitivity testing (FDR-adjusted q < 0.05, Holm correction).

We identified 11 significant associations between SCFA concentrations and bacterial taxa abundance, five of which remained robust after ANCOM-BC2 sensitivity testing (Fig. 10). *Christensenellaceae* exhibited a consistent negative correlation with all major SCFAs. Additional taxa negatively associated with SCFAs included *UBA1819*, *Turicibacter*, *Terrisporobacter*, and *Ruminococcaceae*, specifically in relation to propionic acid. To examine links between microbiota composition and GI barrier integrity, correlations with lipopolysaccharide-binding protein (LBP) levels were analyzed using the same approach; however, no significant associations were observed.

## Discussion

This study provides a comprehensive comparison of clinical, biochemical, anthropometric, psychiatric, and microbiomes in patients with acute and severe and enduring anorexia nervosa relative to healthy controls. While previous research has explored gut microbiota in AN^2–6,11–15^, few studies have differentiated between its acute and chronic forms. A comparison of GI microbiota involvement in both phases of AN may help identify factors contributing to disease persistence. Our findings reveal significant differences in physical health, psychiatric profiles, GI microbiota composition, and interindividual variability, underscoring the complexity and progression of AN and the interplay between physical and psychological factors over time.

Consistent with the recognized physical manifestations of AN, patients with acute and SEAN showed significantly lower body weight, BMI, body fat, and waist and hip circumference compared to HCs, reflecting severe malnutrition. Elevated hyperactivity and greater use of psychotropic medications among AN patients underscore the disorder’s neurobiological and psychiatric complexity^16^. Higher antidepressant use in SEAN patients likely reflects the longer duration of the disease and the increased adult stress reported by SEAN patients may contribute to disordered eating through chronic stress exposure and maladaptive coping^17^ (Tables 1 and S1). Biochemically, AN patients exhibited reduced cholinesterase and fT3 levels, indicating thyroid dysfunction^6,7,18^, along with altered IgA and IgM levels, possibly reflecting GI damage, findings consistent with gut-brain axis disruption in AN^19,20^. Correlations between anthropometric and biochemical markers further support the close relationship between physiological and metabolic alterations in the disorder (Table 1 and Fig. 1).

The psychiatric differences between acute AN and SEAN were notable. As expected, patients with acute AN showed higher EDE-Q and depression scores, reflecting more severe psychological distress in the early stages. In contrast, SEAN patients had lower EDE-Q scores, likely due to entrenched disordered eating patterns replacing extreme restriction. Greater antidepressant use in SEAN suggests a heavier long-term psychological burden (Fig. 2). While anxiety scores were similar, patients with acute AN exhibited more psychological anxiety symptoms. In contrast, SEAN patients showed more somatic anxiety, highlighting the need for tailored treatments addressing both mental and physical symptoms in chronic AN.

Elevated serum I-FABP levels in SEAN patients suggest that prolonged malnutrition may cause intestinal mucosal damage, supported by its correlation with disease duration and lower BMI (Fig. 3). This finding aligns with previous reports of GI dysfunction in AN, which may hinder effective nutritional management and worsen the physical consequences of the disorder^21^.

Despite this, serum levels of amyloid A and calprotectin were low in both AN groups, indicating a suppressed inflammatory response, likely due to malnutrition. However, elevated LBP:sCD14 ratios suggest increased intestinal permeability even without clear systemic inflammation (Fig. 4E). Studies of cytokine levels in AN have found that it is strongly associated with a dysregulated immune system, which is primarily influenced by oxidative stress, chronic psychological stress, and an altered microbiome^22,23^. These findings underscore the complex interplay between malnutrition, immune dysregulation, and GI barrier dysfunction in AN.

Our findings show significant alterations in gut microbiota diversity and composition in both acute AN and SEAN patients compared to HCs. Both patient groups exhibited reduced alpha diversity (Fig. 5A), reflecting a less diverse microbiota, consistent with some prior studies^4,11,13,14,24^, although other studies failed to find different levels of alpha diversity in AN patients vs. healthy individuals^2,6,12,15,25^. Beta diversity analysis revealed distinct microbial profiles, with SEAN patients showing greater interindividual variability (Fig. 5B, C). Similar interindividual variability was demonstrated in a previous study^6^. This increased variability may reflect a progressive loss of microbiota regulation as the illness advances. Supporting this, SEAN patients also showed increased mucosal damage (I-FABP), higher endotoxin exposure (LBP), and reduced inflammatory response (calprotectin), suggesting the gut microbiota may play a role in the chronicity and complexity of AN. These findings suggest that the microbiome undergoes significant changes during the course of AN, possibly contributing to the chronicity and complexity of the disease.

Interestingly, the relative abundance of several bacterial taxa, including E*rysipelatoclostridium*, *Faecalibacterium*, *UBA1819*, *Fusicatenibacter*, *Lachnospiraceae*, and *Bacillota* (formerly *Firmicutes*) bacterium *CAG-56*, differed between AN patients and HCs. These changes in the composition of the gut microbiota may reflect underlying physiological and metabolic adaptations associated with AN. Notably, butyrate-producing bacteria such as *Faecalibacterium* and *Lachnospiraceae* were reduced in AN, consistent with previous findings linking their presence to gut health^25^. The reduced abundance of butyrate-producing bacteria is a common feature of AN^2,6,12^. Lower abundance of *Faecalibacterium* sp. is often described in other diseases, and their presence is likely related to a healthier state of the organism and has been considered for possible therapeutic use. Microbiomes of healthy individuals also contained a higher abundance of *Fusicatenibacter*, which produces formate, lactate, acetate, and succinate as the main products of glucose fermentation. Populations with healthy, high-fiber diets tend to show a higher incidence of *Fusicatenibacter*, and lower levels have been found in people with depression, suggesting a possible link between diet, mental health, and this bacterium^15^.

The *Bacillota* bacterium *CAG-56*, belonging to the *Lachnospiraceae* family, was specifically reduced in acute AN patients compared to HCs, while *UBA1819* from the *Ruminococcaceae* family was elevated in both AN groups. This is supported by a study showing that high-energy diets lead to an decrease in *UBA1819*^26^, and by another study demonstrating a negative correlation between *UBA1819* and subcutaneous fat as well as body weight^27^. The higher occurrence of *Erysipelatoclostridium* in SEAN patients may indicate microbial shifts associated with prolonged malnutrition or altered metabolic pathways (Fig. 6).

Consistent with previous findings, we found that gut microbiota composition was associated with clinical and physiological variables such as BMI, body fat percentage, antidepressant use, antipsychotic use, hormonal contraception, AN type (restrictive vs. purgative), psychiatric disorders, obsessive-compulsive disorder, stress, and fT3 levels (Table 3). It is well established that both the type and quantity of food exert a direct and substantial influence on the composition of the gut microbiota. The use of antidepressants and antipsychotics may alter both the diversity and composition of the gut microbiota, primarily by altering the environment for microbial growth and through their antimicrobial activity^28,29^. Our study also confirmed that the diversity and composition of the gut microbiome are influenced by hormonal factors, including those related to gender, stress, and other endocrine signals^30^.

Our functional predictions using PICRUSt2 indicate significant metabolic alterations primarily in SEAN patients compared to HCs, particularly in amino acid and energy metabolism (Fig. S3). These changes likely reflect long-term adaptations to chronic malnutrition.

Altered oxidative stress and detoxification suggest ongoing cellular stress and damage repair mechanisms, while disruptions in glucose metabolism may relate to prolonged fasting. These findings support our previous observations of oxidative stress, vitamin deficiencies, muscle loss, and reduced ketone body levels in AN, highlighting the metabolic complexity of severe and enduring forms of the disorder^7^.

We observed subtle differences in gut neurotransmitter levels, while significant alterations in SCFA concentrations between HCs and AN patients were observed, which may reflect disturbances in gut-brain interactions. Concentrations of serotonin and tryptophan in stool samples showed no significant differences between HCs and AN patients, although GABA concentrations were slightly elevated in HCs compared to acute AN patients (Fig. 7), which is consistent with previous findings indicating minimal changes in gut-derived neurotransmitters in AN. It appears that the pathology of AN does not substantially disrupt the synthesis of serotonin and other neurotransmitters in the gut, or if it does, the changes are subtle^6^. The limited detection of dopamine, hydroxytryptophan, and kynurenine in stool samples further limits conclusions about their gut-level involvement. In contrast, serum levels of GABA, serotonin, and kynurenine were consistently lower in AN patients, potentially reflecting systemic neurotransmitter dysregulation or altered central signaling (Fig. 8) in AN. The decreased serum neurotransmitter levels may reflect gut-brain axis dysregulation or altered central nervous system function in AN, although the relatively low serum levels of these molecules limit our ability to infer direct links to disease pathology.

Compared to neurotransmitter levels, SCFAs showed more pronounced differences, with HCs exhibiting higher levels of acetic, propionic, butyric, valeric, and hexanoic acids in stool samples (Fig. 7). This supports the idea of microbiome-driven metabolic alterations in AN. These findings are consistent with previous studies showing that SCFAs play a role in maintaining gut health and modulating the gut-brain axis, and their low levels corroborate findings of elevated levels of the intestinal mucosal damage marker I-FABP in the blood (Fig. 3). Lower SCFA levels in AN could indicate dysbiosis or a disturbed gut microbiota composition, both of which have previously been associated with AN^2,6,12,31^.

Interestingly, heptanoic acid levels were higher in HCs than in acute AN patients, with a slight but significant difference between acute AN and SEAN, suggesting its potential as a marker of disease progression or treatment response, though its role remains unclear. In contrast, isobutyric and isovaleric acids showed no significant group differences, indicating limited sensitivity to AN-related metabolic changes. Serum SCFA levels were generally lower than in stool but still showed group differences, particularly for hexanoic, valeric, and isovaleric acids (Fig. 8). These shifts may reflect systemic metabolic alterations, though their low serum concentrations limit interpretation of SCFA dynamics outside the GI tract. Overall, our findings align with previous studies on SCFA changes in AN^31,32^.

The correlation between gut microbiota and metabolites offers intriguing insights into the gut-brain axis in AN. We found a significant negative association between GABA levels and several bacterial taxa such as *Christensenellaceae*, *Ruminococcaceae*, and *Escherichia-Shigella*, suggesting their microbial involvement in GABA metabolism (Fig. 9). Some species, such as *Bifidobacterium*, *Lactobacillus*, and *Bacteroides*, produce GABA via glutamic acid decarboxylase^33^, while others, such as *E. coli*, degrade it through GABA aminotransferase^34^. In our previous study, we found a higher prevalence of *Christensenellaceae* in patients with AN, as well as lower fecal GABA levels. Although we did not observe altered levels of *Christensenellaceae* in this study, we found a negative association between *Christensenellaceae* and GABA levels, which was consistent with our previous study^6^. The bacterial species *Evtepia gabavorous*, belonging to the *Ruminococcaceae* family, has also been described as using GABA for growth^33^, which also supports our results.

A broad range of bacterial taxa correlated with SCFA concentrations, particularly acetic, propionic, and butyric acids. *Christensenellaceae* showed consistent negative correlations with major SCFAs, aligning with its known association with low BMI and reduced SCFA levels in AN patients. Other genera, including *UBA1819*, *Turicibacter*, *Terrisporobacter*, and *Ruminococcaceae*, were also negatively correlated with propionic acid (Fig. 10). The negative correlation between *Christensenellaceae* and SCFA levels is consistent with the finding that *Christensenellaceae* tend to be found in individuals with low BMI; reduced SCFA levels are also observed in AN patients^35^.

These findings underscore the complexity of microbiota-metabolite interactions and suggest that impaired microbial fermentation or dysbiosis in AN may contribute to reduced SCFA production.

This study’s strengths include robust microbiome bioinformatics analysis, despite some limitations. A small sample size may limit generalizability, and neurotransmitter detection was hindered by potential degradation during sample handling. Additionally, the predictive nature of 16S rDNA analysis limits conclusions about functional activity. Still, the results reveal important metabolic trends that warrant further investigation. A key limitation of this study was the high prevalence of constipation among AN patients, which, according to some studies, strongly influences gut microbiota composition^36^. In microbiome studies, the presence of constipation in the majority of patients at the beginning of nutritional therapy obscures the effect of an anorexia diagnosis. A potential solution would be to collect stool samples from patients later in treatment, after bowel function normalizes; this approach was beyond the scope of our study. Finally, while PICRUSt2 provides valuable insights into potential functional differences between groups, it infers metabolic capacity from 16S rRNA gene profiles and does not reflect actual gene expression or metabolite concentrations. Its predictive power is also constrained by the completeness and accuracy of existing reference genomes.

This study underscores significant and progressive alterations in gut microbiota composition, microbial metabolism, and host physiological responses in both acute and chronic forms of AN. Patients with SEAN exhibited greater interindividual variability in microbiota profiles, elevated markers of intestinal barrier dysfunction, and distinct microbial signatures potentially linked to prolonged malnutrition and systemic stress. Notably, reductions in GABA and SCFA levels, alongside specific microbial associations, support the hypothesis of gut-brain axis dysregulation in AN. These findings suggest that gut microbiota disturbances may not only reflect the disease state but also contribute to its persistence, particularly in chronic cases. Integrating microbiome-based diagnostics and therapeutics into clinical practice may offer new avenues for managing and potentially reversing the long-term impacts of AN.

## Methods

### Participants

The study was conducted in accordance with the Declaration of Helsinki and approved by the Ethics Committee of the General University Hospital in Prague. Written informed consent was obtained from all participants.

Eligible participants were women aged 18–40 years. Exclusion criteria included pregnancy, breastfeeding, active infections, severe or chronic diseases (cardiovascular, hematopoietic, hepatic, or renal), recent antibiotic or antimycotic use (within 3 months), or impaired capacity to provide informed consent. Healthy controls (HCs) had no personal or family history of eating or psychiatric disorders and were screened for subclinical symptoms using the SCOFF questionnaire.

The study enrolled 29 women with early-stage disease (less than 3 years since the first clinically significant AN symptoms; inpatients, outpatients, or day-care patients), 33 patients with severe and enduring AN (SEAN; more than 7 years since the first clinically significant AN symptoms; inpatients, outpatients, or day-care patients), and 30 age-matched healthy controls (HCs) (Table 1). Patients were recruited from the Center for Eating Disorders at the Psychiatric Department of the 1st Faculty of Medicine, Charles University, and General University Hospital, Prague. All AN patients were examined at the Center for Eating Disorders located at the Psychiatric Department of the 1st Faculty of Medicine, Charles University and General University Hospital, Prague, CZ. All patients were evaluated by physicians specializing in eating disorders to determine whether they met the Diagnostic and Statistical Manual of Mental Disorders (DSM-5) criteria for AN. This screening was also used to determine the severity of AN (0–5: none, mild, moderate, severe, and extreme). The Exercise and Eating Disorders questionnaire, version 3 (EED19), was used to assess hyperactivity (0–2: none, present, considerable). During refeeding in malnourished patients, fiber intake was gradually introduced, starting at 10–15 g/day and increasing to 21–25 g/day depending on patient tolerance and gastrointestinal condition. Participants were screened for medication use, comorbidities (Table S1), allergy history, and past stressful events (childhood, adolescence, adulthood). AN subtype (restrictive or purgative) was also recorded (Table 1). Healthy controls were recruited from university students, office workers, and local employees.

### Anthropometric measurements and biological sample collection

Anthropometric measurements, including body fat percentage, height, weight, and waist and hip circumference, were obtained via bioimpedance analysis (TANITA, Japan) by trained nursing staff (Table 1). Morning fasting blood samples (Table 1) were collected from the cubital vein by personnel from the psychiatric department. Half were analyzed at the hospital’s central laboratory for standard biochemistry; the remainder was processed at the Institute of Microbiology, with serum aliquots stored at −80 °C. Stool samples were collected the same day and frozen at −80 °C. Prior to collection, participants were asked to refrain from drinking alcohol, coffee, or black tea; not to eat any products containing cocoa, chocolate, nuts, or bananas; and not to take any probiotics.

### Biochemical analysis of blood samples

Blood samples from patients and controls were analyzed for serum levels of triacylglycerols (TAG), cholinesterase, albumin, immunoglobulins (IgA, IgG, IgM), thyroid-stimulating hormone (TSH), free thyroxine (fT4), and free triiodothyronine (fT3) (Table 1). Additional serum markers were assessed using ELISA, including intestinal fatty acid-binding protein (I-FABP), lipopolysaccharide-binding protein (LBP), soluble CD14 (sCD14), calprotectin, and serum amyloid A (SAA).

I-FABP was measured in duplicate using ELISA (HyCult Biotechnology, Netherlands; 1:5 dilution). SAA was measured with a 10-fold dilution (R&D Systems, USA), and LBP and sCD14 with a 100-fold dilution (R&D Systems, USA). Calprotectin (S1008/S1009) was measured using a 300-fold dilution (R&D Systems, USA). All assays followed the manufacturer’s protocols. Absorbance at 450 and 650 nm was read using a Multiskan Ascent Plate Reader (MTX Lab Systems, USA).

### Eating Disorder Evaluation Questionnaire (EDE-Q)

Patients with anorexia nervosa (AN) completed the Eating Disorder Examination Questionnaire version 6.0 (EDE-Q 6.0)^37^, a 28-item self-report instrument derived from the Eating Disorder Examination (EDE)^38^. Of these, 22 items assessed four symptom domains over the past 28 days: (1) restraint, (2) concern with body shape, (3) concern with body weight, and (4) eating concern. Responses were rated on a 7-point Likert scale (0–6). Subscale scores were averaged to produce an overall global score, with higher scores reflecting greater severity of eating disorder psychopathology.

An additional six items evaluated the frequency of behavioral symptoms, such as binge eating, self-induced vomiting, laxative use, and excessive exercise, over the same 28-day period. These items were not included in subscale calculations. All outcome data are visualized using boxplots, with whiskers indicating minimum and maximum values for each subscale and total scores.

### Hamilton Anxiety Rating Scale (HAMA)

Patients with anorexia nervosa (AN) completed the 14-item Hamilton Anxiety Rating Scale (HAMA)^39^ to assess the severity of anxiety symptoms. The questionnaire captures both psychological and somatic dimensions of anxiety. Each item is scored on a scale from 0 (not present) to 4 (severe), yielding a total score ranging from 0 to 56.

Interpretation of total scores is as follows: < 17 indicates mild anxiety, 18–24 mild to moderate anxiety, 25–30 moderate to severe anxiety, > 30 severe anxiety.

### Hamilton Psychiatric Rating Scale for Depression (HAMD)

Depression symptoms in patients with anorexia nervosa (AN) were evaluated using the 17-item version of the Hamilton Depression Rating Scale (HAMD-17)^40,41^ via structured interview conducted by a trained psychiatrist. While a total of 21 items were analyzed, only the first 17 were scored in accordance with HAMD-17 guidelines.

Eight items were rated on a 5-point scale (0–4), corresponding to absent, doubtful, mild, moderate, and severe symptoms. The remaining nine items were scored on a 3-point scale (0–2), reflecting absent, slight/clear, and marked/severe symptoms. Total score interpretations were as follows: < 7 indicates no depression, 7–17 mild depression, 18–24 moderate, depression, > 24 severe depression.

### Statistical analysis

Group age differences were assessed using the Kruskal-Wallis test, followed by Dunn’s post hoc test. Comparisons between anorexia nervosa (AN) subgroups were analyzed using the Mann-Whitney test. Categorical variables, including AN severity/type, stress levels, hyperactivity, allergies, medication usage, and comorbidities, were evaluated using Fisher’s exact test.

Anthropometric, biochemical, and questionnaire-based data were analyzed using two-way ANOVA following Box-Cox transformation (λ = 0.1), with Tukey’s post hoc test applied for pairwise comparisons. Serum biomarkers reflecting inflammation and endotoxin exposure were analyzed via one-way ANOVA, also using Box-Cox transformed values and Tukey’s test. Correlations between variables were explored using Spearman’s rank correlation.

Statistical significance was defined as: *p* < 0.05 (*), *p* < 0.01 (**), *p* < 0.001 (***), *p* < 0.0001 (****). All analyses were performed using GraphPad Prism 8 and RStudio (v2023.6.0.421).

To examine patterns across questionnaire responses, a principal component analysis (PCA) was conducted using five subscales from the EDE-Q (restraint, eating concern, shape concern, weight concern, and total score) and three components from the HAMA (somatic anxiety, psychological anxiety, and total score). Prior to PCA, pairwise Pearson correlation was used to assess multicollinearity and confirm suitability for dimensionality reduction.

### Gut microbiota analysis

Genomic DNA was extracted from stool samples using the ZymoBIOMICS DNA Miniprep Kit (Zymo Research) to enable high-throughput sequencing of the bacterial V3-V4 region of the 16S rRNA gene. Amplicon libraries were generated via a two-step PCR protocol^42^. In the first step, primers S-D-Bact-341-a-A-21 (5′-CCTACGGNGGCWGCAG-3′) and S-D-Bact-0785-a-A-21 (5′-GACTACHVGGGTATCTAATCC-3′) were used to amplify the target region, with additional “tails” incorporated as priming sites for the outer primers^43^. The second step introduced barcoded outer primers containing Illumina sequencing adapters.

PCR amplification was performed using Kapa HiFi HotStart ReadyMix (Roche) with the following cycling conditions: First PCR: 95 °C for 3 min; then 28 cycles of 98 °C for 20 s, 55 °C for 30 s, 72 °C for 30 s; followed by 72 °C for 5 min. Second PCR: 95 °C for 3 min; then 12 cycles of 98 °C for 20 s, 55 °C for 30 s, 72 °C for 30 s; followed by 72 °C for 5 min.

Sequenced samples were prepared in duplicate, each with distinct inline barcodes. The amplicon library was quantified via capillary electrophoresis using the QIAxcel Advanced DNA Screening Kit 2400 (QIAGEN), pooled equimolarly, and purified using SPRIselect magnetic beads (Beckman Coulter).

Amplicons were sequenced using MGIEasy Universal Library Conversion Kits (App-A) on an MGI DNB-SEQ-G400 platform (2 × 300 bp paired-end reads, 600 cycles; MGI, USA) at CEITEC, Brno, Czech Republic.

To assess the integrity of DNA extraction, library preparation, and sequencing performance, ZymoBIOMICS microbial community standards, including evenly and log-distributed DNA and cell mixtures, were used as internal controls (Table S3 and Fig. S5). All controls yielded expected profiles, confirming protocol reliability. DNase-free water controls (Kitom extraction blank and PCR negative control) demonstrated no contamination. However, one water control sample yielded 28 *Bacteroides* sequences, indicating minimal background signal.

### Bioinformatic pipeline

Raw sequencing data were produced in FASTQ format and processed using Skewer^43^ for demultiplexing and trimming. Reads with an expected error rate < 2 were retained as high-quality sequences. These were denoised using DADA2^44^ to generate amplicon sequence variants (ASVs) and construct an abundance matrix.

Chimeric sequences were identified and removed using DADA2’s removeBimeraDenovo function. Taxonomic assignment was performed via the RDP classifier, applying an 80% confidence threshold against the SILVA v138.14 reference database.

To confirm technical reproducibility, Procrustes analysis (*p* = 0.001, R = 0.987) was applied to ASV profiles from sample duplicates. Only ASVs found in both replicates were retained.

The resulting bacterial dataset contained 1893 ASVs, encompassing 1,355,626 high-quality reads, with a mean sequencing depth of 15,582 reads per sample (range: 1,689–80,375).

Microbial functional potential was predicted using the PICRUSt2^45^ pipeline with default parameters. Weighted NSTI scores were consistent across groups (ANOVA *p* = 0.556, F = 0.591). Differential frequencies of predicted enzyme functions were determined using ANCOM-BC2.

### Statistical analyses of microbiome data

All statistical analyses were conducted in RStudio (version 2023.6.0.421). To account for differences in sequencing depth across samples, amplicon sequence variants (ASVs) were rarefied to the minimum sequencing depth prior to analysis.

For alpha diversity, the Shannon index and ASV richness were calculated as response variables. These measures were normalized using Box-Cox transformations and compared between study groups using analysis of variance (ANOVA).

To explore the impact of clinical and demographic factors, such as body mass index (BMI), body fat percentage, and AN subtype, on alpha diversity, predictive variables were first selected via lasso regression. Variables with non-zero coefficients were then included in subsequent linear models.

The relationship between laxative use and Shannon diversity was assessed using linear regression, with laxative use coded as a binary predictor.

Beta diversity was visualized using principal coordinate analysis (PCoA), with dissimilarity matrices computed from Bray-Curtis and Jaccard indices^46^. Group-level differences in microbial composition were assessed via pairwise PERMANOVA using the adonis2 function in the vegan R package.

Interindividual variation within groups was quantified using PERMDISP (betadisper function, vegan package)^46^ to test for differences in dispersion of sample distances.

To investigate the effects of clinical variables on beta diversity, distance-based redundancy analysis (db-RDA) was performed using both dissimilarity metrics as response variables. Forward stepwise model selection was implemented to iteratively add predictors with the strongest associations to the initial null model containing only dissimilarity data.

Differences in bacterial taxa abundance between study groups were evaluated at the genus level using ANCOM-BC2^47^. We performed both a global test to assess overall group differences and a post hoc analysis for pairwise comparisons between groups.

Subsequent ANCOM-BC2 analyses explored associations between microbial taxa and concentrations of individual short-chain fatty acids (SCFAs) or neurotransmitters. To avoid multicollinearity, each biomarker was analyzed independently. As concentrations of SCFAs and neurotransmitters exhibited highly skewed distributions, we applied square root transformations to minimize the influence of high-leverage data points.

All models were statistically adjusted for study group differences, with group affiliation included as a covariate to control for systematic variability. A structural zero sensitivity filter in ANCOM-BC2 was applied to assess robustness against pseudo-count inflation and data sparsity. Taxa that passed this filter were deemed both statistically significant and biologically robust, indicating that observed differences were unlikely to be artifacts of sparse or zero-inflated data.

In line with ANCOM-BC2 developer recommendations, we report these sensitivity analyses to support the reliability and reproducibility of our taxonomic findings.

### Measurement of neurotransmitter levels in stool and serum by LC-MS/MS analysis

Approximately 100 mg of stool was mixed with 1 mL of Milli-Q water (Smart2Pure™ Water Purification System, Thermo Scientific™) and homogenized by vortexing. If less stool was available, the volumes of all reagents were proportionally reduced. The homogenate was centrifuged at 30,000 × g for 10 min at 4 °C, and the resulting supernatant was combined with 1 mL of LC-MS-grade acetonitrile (CHROMASOLV™, Honeywell). The mixture was then incubated at −20 °C for 30 min and subjected to a second centrifugation (30,000 × g, 4 °C, 10 min). Finally, 1 mL of supernatant was collected for LC-MS/MS analysis.

For serum samples, 50 µL was mixed with 200 µL of acetonitrile:methanol (3:5, LC-MS grade) and incubated at −20 °C for 30 min. Samples were centrifuged at 7,700 × g for 10 min at 4 °C, and the supernatant was used for LC-MS/MS.

Targeted analysis was performed using an Agilent Infinity 1260 liquid chromatograph coupled to an Agilent 6470 LC/TQ mass spectrometer. Analytes were separated on a Kinetex Polar C18 analytical column (2.6 μm, 3 mm × 100 mm) with a SecurityGuard Polar C18 precolumn (2.6 μm, 3 mm × 2 mm; Phenomenex), both maintained at 40 °C.

Elution was carried out using a gradient program with mobile phases: Phase A: 0.1% formic acid in Milli-Q water (LC-MS grade, Honeywell); Phase B: 0.1% formic acid in acetonitrile (LC-MS grade, CHROMASOLV™, Honeywell). Gradient steps were as follows:

(time [min], % phase B): 0/0 → 1/0 → 5/20 → 6/100 → 8/100 → 8.5/0 → 9/0

The mobile phase flow rate was set to 0.6 mL/min, and 2.0 μL of sample was injected per run. Matrix effects were minimized via automatic standard additions, whereby each sample was injected alongside its respective internal standard.

Mass spectrometric parameters, including ion transitions, were optimized using MassHunter Workstation Optimizer and Source Optimizer software (version 10.0, SR1; Agilent). Instrument settings included a gas temperature of 210 °C and a gas flow rate of 12 L/min. All analytical standards were obtained from Sigma-Aldrich®. Further technical details, including calibration and validation metrics, are summarized in Table S4.

### Measurement of short-chain fatty acids in stool and serum by GC-MS analysis

Approximately 200 mg of stool (or less, with proportional volume adjustment) was mixed with 1 mL of Milli-Q water (Smart2Pure™ Water Purification System, Thermo Scientific™), homogenized by vortexing, and centrifuged at 30,000 × g for 10 min at 4 °C. A total of 500 μL of the supernatant was then combined with 50 μL of 30% hydrochloric acid (Lachner, Czech Republic) and 600 μL of stabilized diethyl ether (VWR Chemicals BDH®, USA).

After 3 min of vortexing at 4 °C, the mixture was centrifuged again (30,000 × g for 10 min at 4 °C), and the diethyl ether phase was carefully transferred into a pre-weighed 2 mL iron vial. The extraction was performed twice, and the combined organic phases were weighed prior to GC-MS analysis.

For serum extraction, 500 μL of sample (adjusted with Milli-Q water if necessary) was mixed with 50 μL of 30% hydrochloric acid (Lachner, Czech Republic) and 600 μL of stabilized diethyl ether (VWR Chemicals BDH®, USA). The mixture was vortexed for 3 min at 4 °C, followed by centrifugation at 6000 × g for 4 min at 4 °C.

The resulting diethyl ether phase was carefully transferred into a pre-weighed 2 mL vial. The extraction was repeated twice, and the combined organic fractions were weighed prior to GC-MS analysis.

Targeted GC-MS analysis was performed using a Varian 450-GC gas chromatograph coupled with a Varian 240-MS mass spectrometer (Varian Inc., USA). Chromatographic separation was achieved using a DB-WAXETR column (0.25 μm film thickness; 30 m × 0.25 mm internal diameter), fitted with helium as the carrier gas at a constant flow rate of 1.4 mL/min.

Each sample (1 μL) was injected in split/splitless mode, with a split ratio of 1:50 activated after 1 min. The oven temperature program was as follows: 50 °C for 1 min, ramp to 140 °C at 20 °C/min, ramp to 150 °C at 10 °C/min (hold 1 min), ramp to 180 °C at 15 °C/min, final ramp to 230 °C at 20 °C/min (hold 1 min), total run time: 13.5 min.

Operating temperatures for the injector, electron ionization (EI) source, and transfer line were 230 °C, 250 °C, and 280 °C, respectively. A solvent delay of 3 min was applied.

Mass spectra were acquired in total ion current (TIC) mode from 3 to 13.5 min, across a mass range of 30–300 m/z. Selected ion monitoring (SIM) mode was used to detect target analytes based on Q1 mass transitions derived from Zhu et al.^48^. Samples were diluted 10-fold and quantified using external calibration curves.

Detection limits ranged from 0.1 to 25 µg/g for stool and 0.01 to 25 µg/mL for serum, depending on analyte-specific characteristics. Measured concentrations were subsequently converted to μg/mL of serum for comparative analysis.

## Supplementary information

Supplemental information

## Data Availability

Sequencing data are archived in the European Nucleotide Archive under project PRJEB77672. Accession numbers with metadata for each sample and R scripts are available at the GitHub repository (https://github.com/JanetJezkova/Gut-microbiota-in-patients-with-Anorexia-Nervosa---acute-vs.-chronic-patients). The data from this study are available in the Open Research Repository Zenodo: doi: 10.5281/zenodo.15102456.
