# Supplementary material for: Microbiome and metabolic disruption in acute vs. severe and enduring anorexia nervosa
Source: NPJ Biofilms Microbiomes. 2025 Nov 26;11:217. doi: 10.1038/s41522-025-00847-y (PMC12657967; doi:10.1038/s41522-025-00847-y)
Supplement: Supplementary file 1 — Supplemental information [file 41522_2025_847_MOESM1_ESM.pdf]

Microbiome and Metabolic Disruption in Acute vs. Severe and Enduring Anorexia Nervosa

Petra Prochazkova[a*], Janet Jezkova[a,b], Radka Roubalova[a], Katerina Zadakova[a] Kristyna Coufalova[a], Gabriela Kubisova[a], Jakub Kreisinger[c], Jaroslav Semerad[a,d], Alena Nehasilova[a], Tomas Cajthaml[a,d], Helena Tlaskalova-Hogenova[a], Petra Holanova[e], Alena Lambertova[e], and Hana Papezova[e]

[a]Institute of Microbiology of the Czech Academy of Sciences, Prague, Czech Republic
[b]First Faculty of Medicine, Charles University, Prague, Czech Republic
[c]Faculty of Science, Department of Zoology, Charles University, Prague, Czech Republic
[d]Institute for Environmental Studies, Faculty of Science, Charles University, Czech Republic
[e]Department of Psychiatry, First Faculty of Medicine, Charles University and General University Hospital in Prague, Czech Republic

Supplementary data

Table S1. Proportional representation of comorbid disorders or features in acute AN and SEAN.

Comorbid disorder/feature	Acute AN	SEAN	Acute AN vs. SEAN p-value
Alcohol-, Sedative-, Hypnotic-, or Anxiolytic-related disorders	0/29 (0%)	4/33 (12.12%)	0.12
Manic episode	1/29 (3.45%)	0/33 (0%)	0.47
Major depressive disorder	8/29 (27.59%)	9/33 (27.27%)	1
Dysthymic disorder	3/29 (10.34%)	3/33 (9.09%)	1
Agoraphobia	0/29 (0%)	2/33 (6.06%)	0.49
Social anxiety disorder	3/29 (10.34%)	4/33 (12.12%)	1
Panic disorder	5/29 (17.24%)	2/33 (6.06%)	0.24
Generalized anxiety disorder	5/29 (17.24%)	4/33 (12.12%)	0.72
Mixed anxiety and depressive disorders	6/29 (20.69%)	9/33 (27.27%)	0.77
Obsessive-compulsive disorder	1/29 (3.45%)	3/33 (9.09%)	0.62
Mixed obsessional thoughts and acts	0/29 (0%)	5/33 (15.15%)	0.12
Post-traumatic stress disorder	2/29 (6.9%)	0/33 (0%)	0.21
Persistent somatoform pain disorder	1/30 (3.45%)	0/33 (0%)	0.47
Emotionally unstable personality disorders	2/29 (6.9%)	1/33 (3.03%)	0.60
Mixed personality disorders	0/29 (0%)	1/33 (3.03%)	1
Suicidality	9/29 (31.03%)	6/33 (18.18%)	0.37

Differences between groups were assessed using Fisher's exact test. SEAN - severe and enduring AN. All comparisons were non-significant.

Table S2. Full names of predicted enzyme functions.

Names generated by PICTRUSt2	Names used in publication
[1] "3-dehydro-L-gulonate 2-dehydrogenase"	3-dehydro-L-gulonate 2-dehydrogenase
[2] "Dihydrokaempferol 4-reductase"	Dihydrokaempferol 4-reductase
[3] "EC:1.1.1.26 Glyoxylate reductase"	Glyoxylate reductase
[4] "EC:1.1.1.287 D-arabinitol dehydrogenase (NADP(+))"	D-arabinitol dehydrogenase
[5] "EC:1.1.1.289 Sorbose reductase"	Sorbose reductase
[6] "EC:1.1.1.304 Diacetyl reductase ((S)-acetoin forming)"	Diacetyl reductase
[7] "EC:1.1.1.310 (S)-sulfolactate dehydrogenase"	Sulfolactate dehydrogenase
[8] "EC:1.1.1.350 Ureidoglycolate dehydrogenase (NAD(+))"	Ureidoglycolate dehydrogenase
[9] "EC:1.1.1.47 Glucose 1-dehydrogenase (NAD(P)(+))"	Glucose 1-dehydrogenase
[10] "EC:1.1.1.76 (S,S)-butanediol dehydrogenase"	Butanediol dehydrogenase
[11] "EC:1.1.1.88 Hydroxymethylglutaryl-CoA reductase"	Hydroxymethylglutaryl-CoA reductase
[12] "EC:1.1.3.21 Glycerol-3-phosphate oxidase"	Glycerol-3-phosphate oxidase
[13] "EC:1.13.11.2 Catechol 2,3-dioxygenase"	Catechol 2,3-dioxygenase
[14] "EC:1.16.1.1 Mercury(II) reductase"	Mercury(II) reductase
[15] "EC:1.2.1.58 Phenylglyoxylate dehydrogenase (acylating)"	Phenylglyoxylate dehydrogenase
[16] "EC:1.2.1.76 Succinate-semialdehyde dehydrogenase (acetylating)"	Succinate-semialdehyde dehydrogenase
[17] "EC:1.2.1.8 Betaine-aldehyde dehydrogenase"	Betaine-aldehyde dehydrogenase
[18] "EC:1.3.1.31 2-enoate reductase"	2-enoate reductase
[19] "EC:1.3.1.33 Protochlorophyllide reductase"	Protochlorophyllide reductase
[20] "EC:1.5.1.28 Opine dehydrogenase"	Opine dehydrogenase
[21] "EC:1.5.3.1 Sarcosine oxidase"	Sarcosine oxidase
[22] "EC:1.6.99.1 NADPH dehydrogenase"	NADPH dehydrogenase
[23] "EC:2.1.1.265 Tellurite methyltransferase"	Tellurite methyltransferase
[24] "EC:2.3.1.118 N-hydroxyarylamine O-acetyltransferase"	N-hydroxyarylamine O-acetyltransferase
[25] "EC:2.3.1.12 Dihydrolipoyllysine-residue acetyltransferase"	Dihydrolipoyllysine-res. acetyltransferase
[26] "EC:2.3.1.203 UDP-N-acetylbacillosamine N-acetyltransferase"	UDP-N-acetylbacillosamine N-acetyltrans.
[27] "EC:2.3.1.82 Aminoglycoside 6-N-acetyltransferase"	Aminoglycoside 6-N-acetyltransferase
[28] "EC:2.3.2.18 glycyltransferase"	Glycyltransferase
[29] "EC:2.4.1.144 Beta-1,4-mannosyl-glycoprotein 4-beta-N-acetylglucosaminyltransferase"	β-mann-gp-4-β-N-acetylglucosaminyltrans.
[30] "EC:2.4.1.208 Diglucosyl diacylglycerol synthase (1,2-linking)"	Diglucosyl diacylglycerol synthase
[31] "EC:2.4.1.216 Trehalose 6-phosphate phosphorylase"	Trehalose 6-phosphate phosphorylase

[32] "EC:2.4.1.247 Beta-D-galactosyl-(1->4)-L-rhamnose phosphorylase"	β-D-galactosyl-L-rhamnose phosphorylase
[33] "EC:2.4.1.64 Alpha,alpha-trehalose phosphorylase"	α-trehalose phosphorylase
[34] "EC:2.5.1.26 Alkylglycerone-phosphate synthase"	Alkylglycerone-phosphate synthase
[35] "EC:2.7.1.100 S-methyl-5-thioribose kinase"	S-methyl-5-thioribose kinase
[36] "EC:2.7.1.146 ADP-specific phosphofructokinase"	ADP-specific phosphofructokinase
[37] "EC:2.7.1.147 ADP-specific glucokinase"	ADP-specific glucokinase
[38] "EC:2.7.1.184 Sulfofructose kinase"	Sulfofructose kinase
[39] "EC:2.7.1.55 Allose kinase"	Allose kinase
[40] "EC:2.7.1.58 2-dehydro-3-deoxygalactonokinase"	2-dehydro-3-deoxygalactonokinase
[41] "EC:2.7.1.95 Kanamycin kinase"	Kanamycin kinase
[42] "EC:2.7.7.83 UDP-N-acetylgalactosamine diphosphorylase"	UDP-N-acetylgalactosamine diphos.
[43] "EC:3.1.1.17 Gluconolactonase"	Gluconolactonase
[44] "EC:3.2.1.11 Dextranase"	Dextranase
[45] "EC:3.2.1.170 Mannosylglycerate hydrolase"	Mannosylglycerate hydrolase
[46] "EC:3.2.1.31 Beta-glucuronidase"	β-glucuronidase
[47] "EC:3.2.1.40 Alpha-L-rhamnosidase"	α-L-rhamnosidase
[48] "EC:3.2.1.64 2,6-beta-fructan 6-levanbiohydrolase"	2,6-β-fructan 6-levanbiohydrolase
[49] "EC:3.3.1.1 Adenosylhomocysteinase"	Adenosylhomocysteinase
[50] "EC:3.4.11.19 D-stereospecific aminopeptidase"	D-stereospecific aminopeptidase
[51] "EC:3.4.11.7 Glutamyl aminopeptidase"	Glutamyl aminopeptidase
[52] "EC:3.4.14.11 Xaa-Pro dipeptidyl-peptidase"	Pro-dipeptidyl-peptidase
[53] "EC:3.5.1.49 Formamidase"	Formamidase
[54] "EC:3.5.1.68 N-formylglutamate deformylase"	N-formylglutamate deformylase
[55] "EC:3.5.2.14 N-methylhydantoinase (ATP-hydrolyzing)"	N-methylhydantoinase
[56] "EC:3.5.3.26 (S)-ureidoglycine aminohydrolase"	Ureidoglycine aminohydrolase
[57] "EC:3.5.99.3 Hydroxydechloroatrazine ethylaminohydrolase"	Hydroxydechloroatrazine ethylaminohydr.
[58] "EC:3.5.99.7 1-aminocyclopropane-1-carboxylate deaminase"	1-aminocycloprop.-1-carboxylate deam.
[59] "EC:4.1.2.52 4-hydroxy-2-oxoheptanedioate aldolase"	4-hydroxy-2-oxoheptanedioate aldolase
[60] "EC:4.2.1.144 3-amino-5-hydroxybenzoate synthase"	3-amino-5-hydroxybenzoate synthase
[61] "EC:4.2.1.28 Propanediol dehydratase"	Propanediol dehydratase
[62] "EC:4.2.1.5 Arabinonate dehydratase"	Arabinonate dehydratase
[63] "EC:4.2.1.83 4-oxalmesaconate hydratase"	4-oxalmesaconate hydratase
[64] "EC:4.2.2.17 Inulin fructotransferase (DFA-I-forming)"	Inulin fructotransferase
[65] "EC:4.4.1.15 D-cysteine desulfhydrase"	D-cysteine desulfhydrase
[66] "EC:4.4.1.25 L-cysteate sulfo-lyase"	L-cysteate sulfo-lyase

[67] "EC:5.1.3.7 UDP-N-acetylglucosamine 4-epimerase"	UDP-N-acetylglucosamine 4-epimerase
[68] "EC:5.1.99.3 Allantoin racemase"	Allantoin racemase
[69] "EC:5.2.1.1 Maleate isomerase"	Maleate isomerase
[70] "EC:5.3.1.29 Ribose 1,5-bisphosphate isomerase"	Ribose 1,5-bisphosphate isomerase
[71] "EC:5.3.2.5 2,3-diketo-5-methylthiopentyl-1-phosphate enolase"	Methylthiopentyl-1-phosphate enolase
[72] "EC:5.3.3.14 Trans-2-decenoyl-[acyl-carrier-protein] isomerase"	Trans-2-decenoyl-isomerase
[73] "EC:6.1.2.1 D-alanine--(R)-lactate ligase"	D-alanine-lactate ligase
[74] "EC:6.3.2.7 UDP-N-acetylmuramoyl-L-alanyl-D-glutamate--L-lysine ligase"	UDP-acetylm.-D-glutamate-L-lysine ligase
[75] EC:3.2.2.23 DNA-formamidopyrimidine glycosylase	DNA-formamidopyrimidine glycosylase
[76] EC:3.2.2.20 DNA-3-methyladenine glycosylase I	DNA-3-methyladenine glycosylase I

Table S3. The original microbial community standards' compositions (ZymoBIOMICS^{TM}) compared to the obtained sequencing data.

**Microbial community standard I**	**gDNA %**	**16S %**	**obtained 16S %**
Pseudomonas aeruginosa	12	4.2	4.0
Escherichia coli	12	10.1	18.8
Salmonella enterica	12	10.4	8.8
Lactobacillus fermentum	12	18.4	9.2
Enterococcus faecalis	12	9.9	8.9
Staphylococcus aureus	12	15.5	9.5
Listeria monocytogenes	12	14.1	16.6
Bacillus subtilis	12	17.4	23.8
Saccharomyces cerevisiae	2	NA	NA
Cryptococcus neoformans	2	NA	NA

**Microbial community standard II**	**gDNA %**	**16S %**	**obtained 16S %**
Listeria monocytogenes	89.1	95.9	89.5
Pseudomonas aeruginosa	8.9	2.8	7.9
Bacillus subtilis	0.89	1.2	2.3
Saccharomyces cerevisiae	0.89	NA	NA
Escherichia coli	0.089	0.069	0.00
Salmonella enterica	0.089	0.07	0.00
Lactobacillus fermentum	0.0089	0.012	0.00
Enterococcus faecalis	0.0089	0.00067	0.00
Cryptococcus neoformans	0.00089	NA	NA
Staphylococcus aureus	0.00089	0.0001	0.00

**Microbial community DNA standard I**	**gDNA %**	**16S %**	**obtained 16S %**
Pseudomonas aeruginosa	12	4.2	4.8
Escherichia coli	12	10.1	12.0
Salmonella enterica	12	10.4	11.1
Lactobacillus fermentum	12	18.4	11.1
Enterococcus faecalis	12	9.9	4.8
Staphylococcus aureus	12	15.5	13.8
Listeria monocytogenes	12	14.1	18.4
Bacillus subtilis	12	17.4	20.6
Saccharomyces cerevisiae	2	NA	NA
Cryptococcus neoformans	2	NA	NA

**Microbial community DNA standard II**	**gDNA %**	**16S %**	**obtained 16S %**
Listeria monocytogenes	89.1	95.9	95.8
Pseudomonas aeruginosa	8.9	2.8	3.6
Bacillus subtilis	0.89	1.2	0.6
Saccharomyces cerevisiae	0.89	NA	NA
Escherichia coli	0.089	0.069	0.00
Salmonella enterica	0.089	0.07	0.00
Lactobacillus fermentum	0.0089	0.012	0.00

Enterococcus faecalis	0.0089	0.00067	0.00
Cryptococcus neoformans	0.00089	NA	NA
Staphylococcus aureus	0.00089	0.0001	0.00

Table S4. The LC-MS/MS parameters.

Compound	Precursor ion	Quantifier ion	Qualifier ion	Polarity	Cell accelerator voltage (V)	Fragmentor (eV)	Quantifier collision energy (eV)	Qualifier collision energy (eV)	Standard addition (ppb)
**Dopamine**	154.1	90.9	136.8	pos	4	72	26	10	50, 250, 1250
**GABA**	104.1	45.2	87.1	pos	4	50	30	10	5, 25, 125
**Hydroxy-tryptophan**	221.1	204	162	pos	4	90	10	18	10, 50, 250
**Kynurenine**	209.2	94.1	146	pos	4	70	14	26	10, 50, 250
**Serotonin**	177.1	160	115	pos	4	50	10	34	50, 250, 1250

GABA – gamma aminobutyric acid; pos – positive.

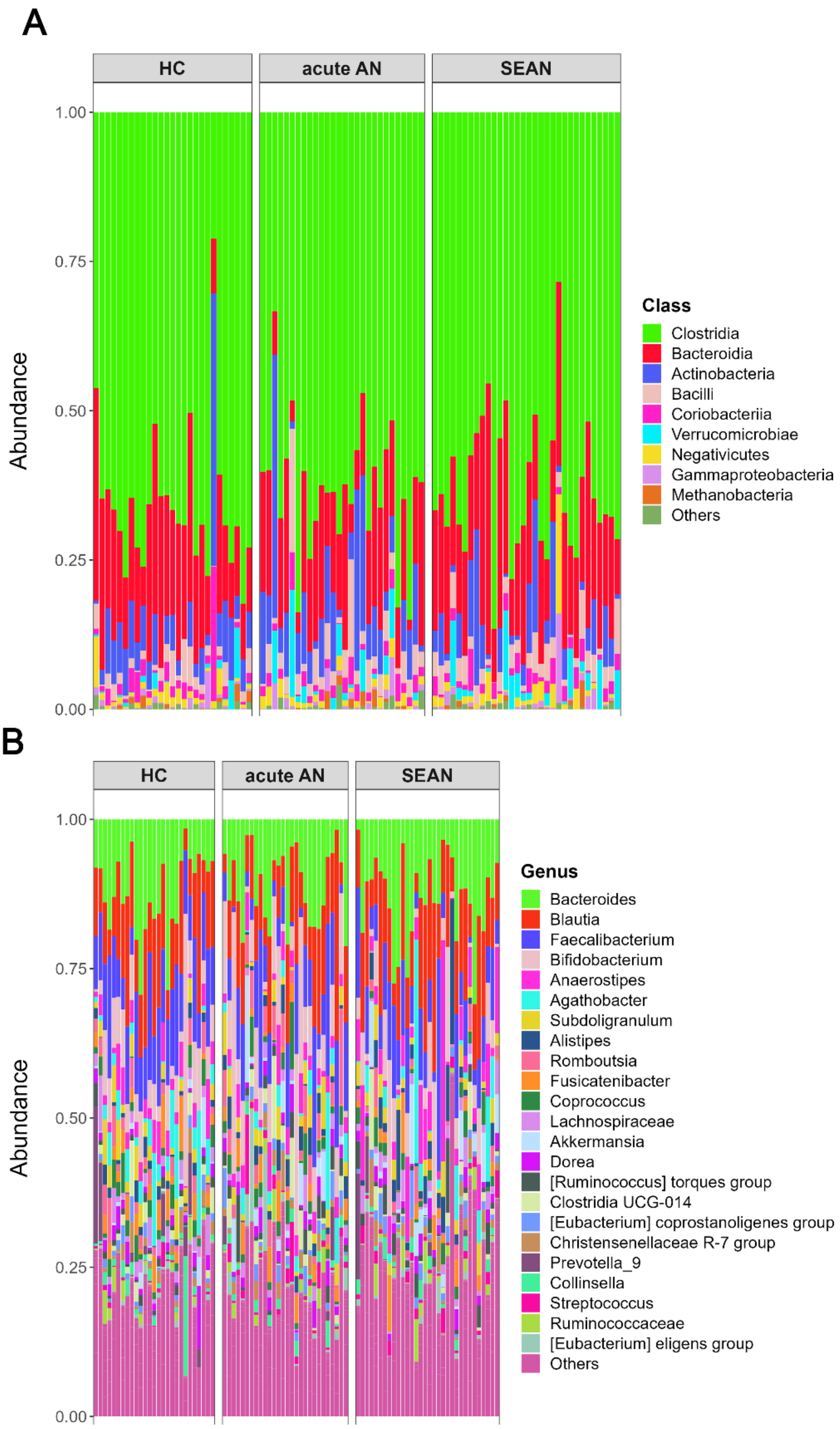

Fig. S1. Proportions of the dominant A) bacterial classes and B) genera (represented by > 1% of reads) in the three groups studied. HC – healthy controls; SEAN – severe and enduring AN.

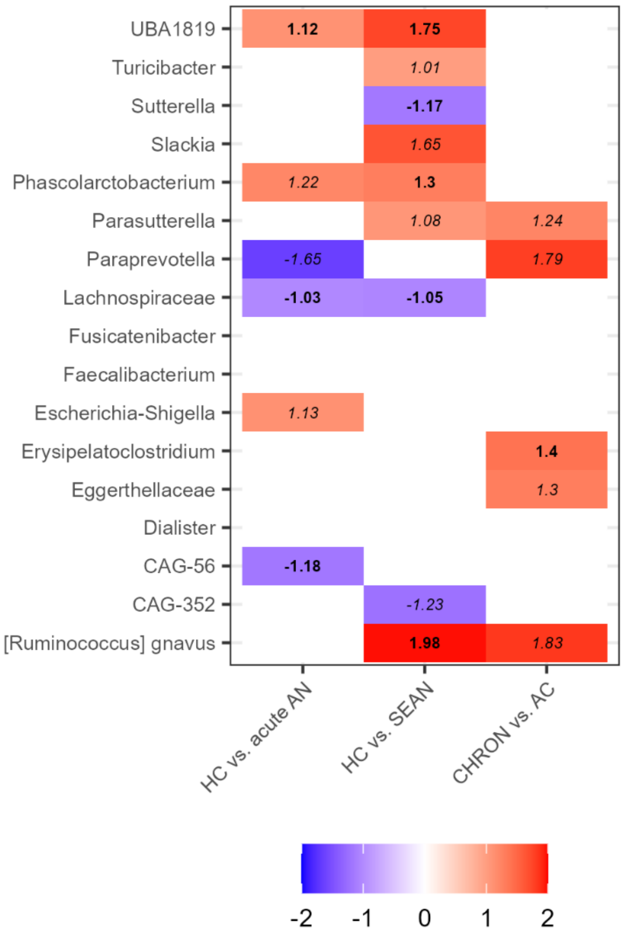

Fig. S2. Differential abundance analysis at the genus level. The heatmap shows the bacterial genera whose abundance varied between the study groups based on ANCOM-BC2. All genera identified by the global test are shown. Pairwise post-hoc comparisons were performed, and the resulting log-fold changes are indicated by the color scale and numerical values within the cells. The color is white if the corresponding pairwise test was not significant. The numbers are printed in italics if the pairwise test did not pass the sensitivity analysis; otherwise, they are printed in bold.

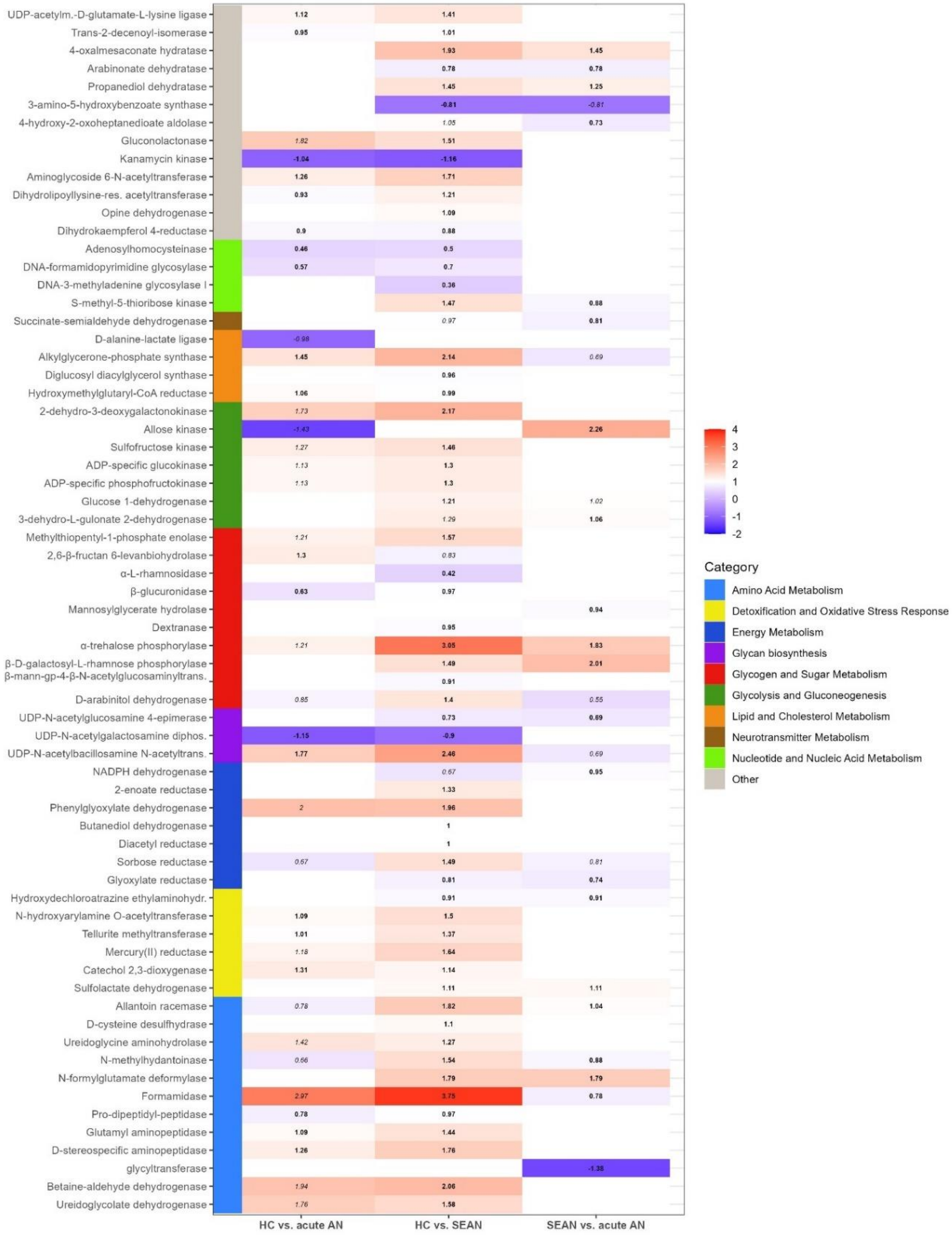

Fig. S3. Differences in the relative abundances of the predicted enzyme functions between the studied groups based on ANCOM-BC2 analyses (FDR < 0.05). The predicted enzyme functions are manually grouped into hypothetical categories based on their biological functions, independent of KEGG or other pathways databases. White indicates non-significant corresponding pairwise tests. Italics indicate that the pairwise test did not pass the ANCOM-BC2 sensitivity test; otherwise, numbers are shown in bold type. HC – healthy controls; SEAN – severe and enduring AN. The full names of predicted enzyme functions with EC numbers are listed in Table S2.

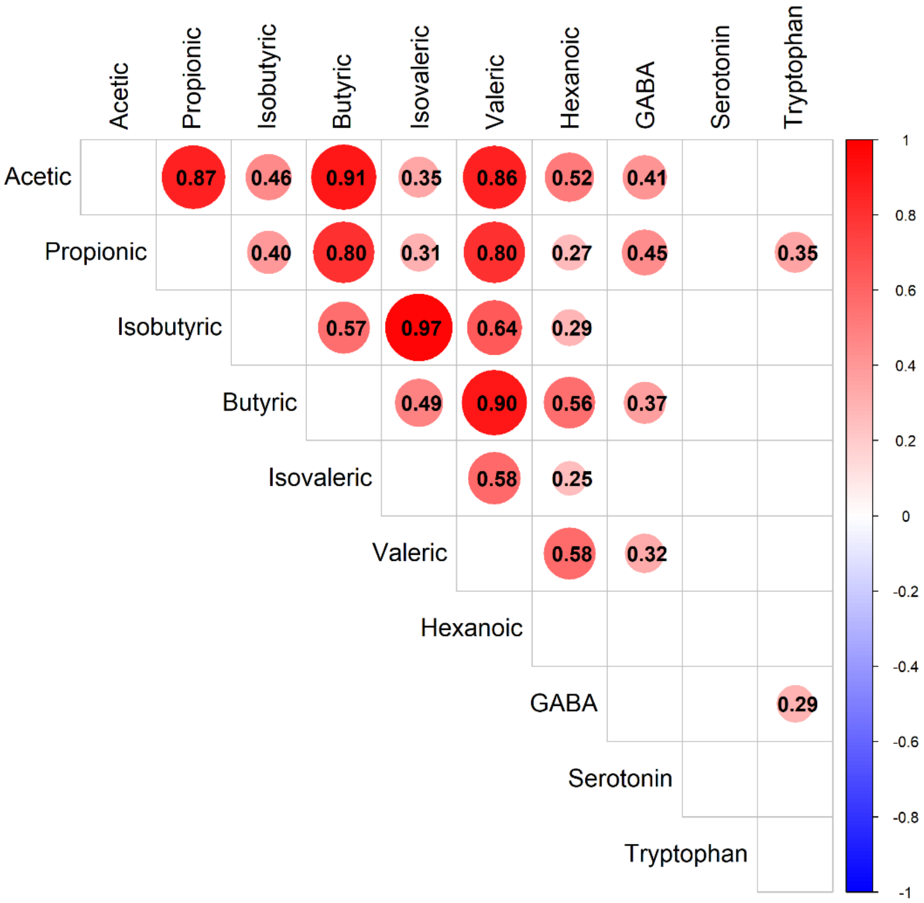

Fig. S4. Correlations between SCFAs and neurotransmitter levels were analyzed with the Spearman correlation coefficient. Only significant correlations are shown.

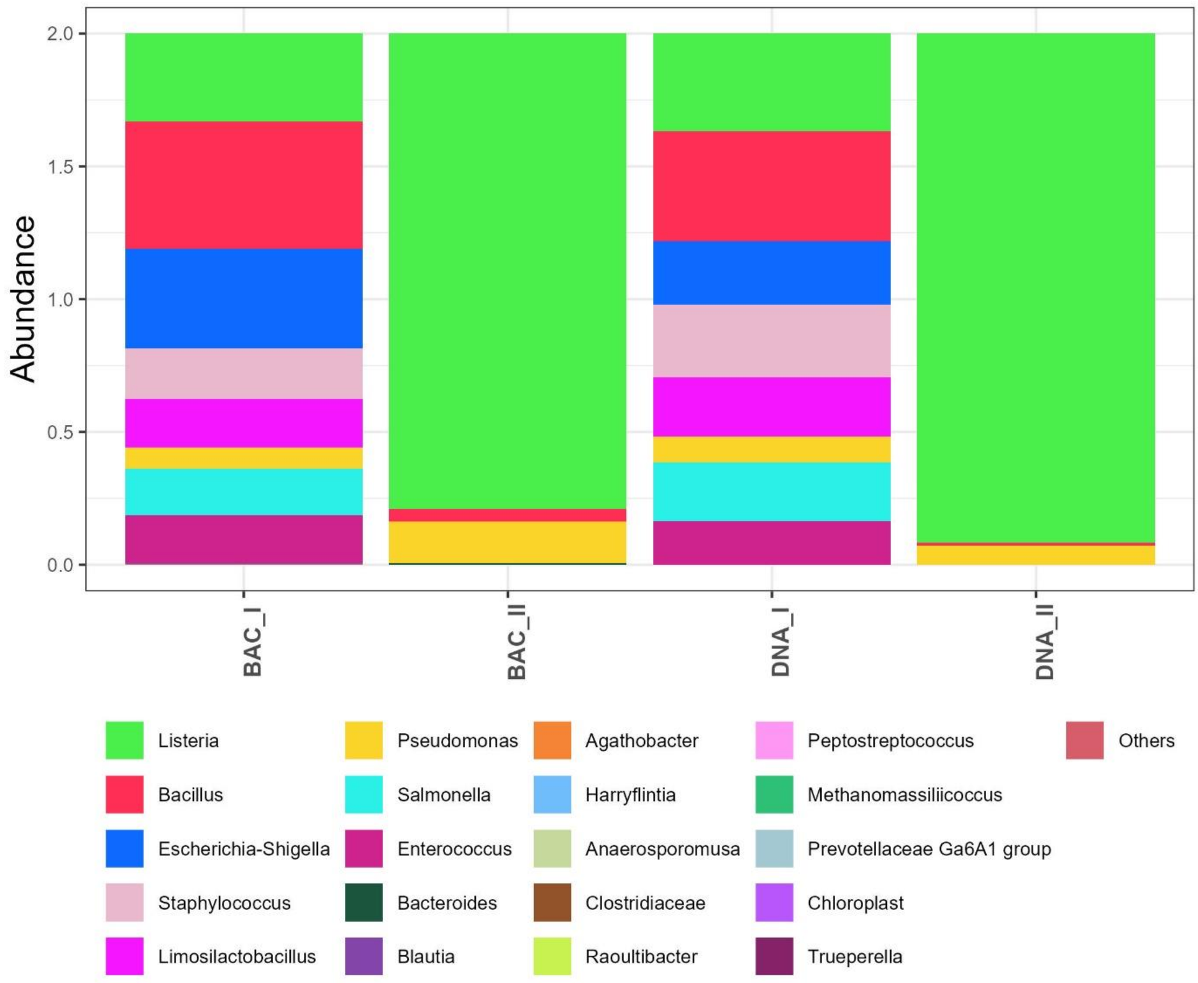

Fig S5. Microbiome analysis of microbial community standards. Bar plots show relative abundances of microbial community standards (ZymoBIOMICS^{TM}). BAC_I – microbial community standard I, BAC_II – microbial community standard II, DNA_I – microbial community DNA standard I, DNA_II – microbial community DNA standard II.
